# How Do Polish Students Manage Emotional Distress during the COVID-19 Lockdown? A Web-Based Cross-Sectional Study

**DOI:** 10.3390/jcm10214964

**Published:** 2021-10-26

**Authors:** Ryszard Sitarz, Alicja Forma, Kaja Karakuła, Dariusz Juchnowicz, Jacek Baj, Jacek Bogucki, Hanna Karakuła-Juchnowicz

**Affiliations:** 1 Chair and I Department of Psychiatry, Psychotherapy and Early Intervention, Medical University of Lublin, 20-439 Lublin, Poland; hannakarakulajuchnowicz@umlub.pl; 2 Psychiatry Student Research Group I Department of Psychiatry, Psychotherapy and Early Intervention, Medical University of Lublin, 20-439 Lublin, Poland; formaalicja@gmail.com (A.F.); kaja.karakula@gmail.com (K.K.); 3 Department of Psychiatric Nursing, Medical University of Lublin, 20-093 Lublin, Poland; dariusz.juchnowicz@umlub.pl; 4 Chair and Department of Anatomy, Medical University of Lublin, 20-090 Lublin, Poland; jacekbaj@umlub.pl; 5 Chair and Department of Organic Chemistry, Faculty of Pharmacy, Medical University of Lublin, 20-093 Lublin, Poland; jacekbogucki@umlub.pl

**Keywords:** COVID-19, SARS-CoV-2, pandemic, emotional distress, depression, anxiety, stress, DASS-21, coping strategies, Brief-COPE

## Abstract

Choices regarding coping strategies during the COVID-19 pandemic outbreak may imply the development as well as the severity of emotional disorders. The aim of this web-based cross-sectional study was to: (1) assess the coping strategies for stress in a population of Polish students and (2) evaluate the impact of those strategies on the severity of depression, stress, and anxiety symptoms during the COVID-19 lockdown. To evaluate emotional distress, we used the DASS-21 scale and coping strategies Brief-COPE Inventory. The study included 2172 respondents (73% female, 27% male) with a mean age of 22.1 ± 2.2. Students more frequently chose stress coping strategies belonging to the ‘approach’ coping strategies (M = 29.60 ± 6.89) compared to ‘avoidant’ coping strategies (M = 22.82 ± 5.78). The intensification of distress in women caused a turn to religion (*p* = 0.001), while men used substances (*p* < 0.001) and a sense of humor (*p* < 0.001). Medical students coped best with emotional distress, which is very encouraging for their future profession. The highest level of DASS total score was associated with the usage of avoidant coping strategies, prior use of psychiatric or psychological support, and loneliness. Planning interventions to prevent emotional disorders in students requires the identification of factors contributing to increased emotional distress.

## 1. Introduction

The novel Coronavirus Disease 2019 (COVID-19), which was officially announced as a pandemic more than one year ago (11 March 2020), remains a huge challenge, particularly for frontline workers, having a significant impact not only on the physical, but also on the mental health of the entire population [1]. According to current global statistics, there are more than 233 million confirmed cases, along with a total number of more than 4 million deaths worldwide, while in Poland there are more than 2 million confirmed cases and more than 75 thousand deaths confirmed (as of 3 October 2021, the World Health Organization (WHO)) [2]. The very wide spectrum of clinical manifestations, as well as the long-term effects associated with the severe acute respiratory syndrome coronavirus-2 (SARS-CoV-2) infection, has continually aroused concern and anxiety among the public [3].

Like many countries [4,5,6,7], Poland has implemented certain relatively effective restrictions to gain control of the pandemic. A sudden outbreak of COVID-19 has forced the government to introduce certain restrictions to prevent viral transmission and minimize the risk of potential infections. In Poland, the lockdown was announced on 10 March 2020, and the restrictions have been continually changing depending on the ongoing situation, and especially on the number of new and confirmed cases, as well as fatal cases. Such quick implementation of strict restrictions, limited information about the virus and disease itself, uncertainty about further recommendations and potential restrictions introduced by the government, continuous need to stay at home, limited contact with family members/friends, fear about future education, as well as limited medical assistance (primarily limited to those with SARS-CoV-2-positive infection or emergency) are only a few of the major issues that significantly changed the way of living of the general population in Poland. These, in consequence, have had a significant impact on mental health and daily well-being.

The pandemic affects all individuals regardless of age or gender; however, young adults remain one of the most susceptible to the negative consequences of the pandemic in the general population as they represent the lowest levels of psychological health during the ongoing situation [8,9]. Students were reported to be the most vulnerable to emotional distress, and this group is characterized by some of the highest depression, anxiety, and stress levels because of the COVID-19 pandemic [10,11,12,13,14]. The ongoing pandemic might either induce psychological/psychiatric disorders or exaggerate already existing ones [14,15]. Even without the COVID-19 pandemic, suicide and depression rates amongst students are reported to be increasing alarmingly nowadays [16,17]. Generally, university studies might constitute a stressful period in students’ lives since they remain in a transition period leading to independent, adult life. Factors associated with the COVID-19 pandemic (fear of infection, insufficient knowledge about the SARS-CoV-2 virus at the beginning of the pandemic, implementation of the strict restrictions by the governments, social isolation, etc.) constitute additional factors that could trigger emotional disturbances for students during the ongoing pandemic.

According to the transactional model of stress and coping by Lazarus and Folkman (1984), psychological stress is closely associated with the person and the environment; the model includes two major phases: (1) cognitive appraisal and (2) coping [18]. Therefore, the stress-coping adjustment process is crucial in this group to alleviate symptoms and provide better general well-being. Lazarus’ classic transactional approach emphasizes the importance of analyzing individuals or subjectively evaluating stressful events that take place in their environment. Thus, it can be assumed that an individual’s interpretation of the environment is the main determinant in triggering a stress response in response to a stressor [19,20]. A crucial matter regarding Lazarus’ approach is taking into account two elements: the question of evaluation (i.e., what will be a burden for a given person) and the type of effort put in to deal with the requirements. In the last version of the understanding theory of stress by Lazarus and Folkman (1986), comprehending stress as a clearly relational concept is proposed [21]. This means that stress is not defined as a specific type of external stimulation or a specific pattern of physiological, behavioral, or subjective responses, but rather as a kind of relationship between individuals and their environment. It is this relationship that will determine whether the available resources prove to be sufficient and effective in coping by activating various types of activities and organizing them into coping strategies [18]. Certain types of stress are embedded in various types of so-called basic emotions [22]. We assumed, following Lazarus [22], that depression can be treated as one of the underlying distress emotions. Previous studies suggest that depression is one of the main psychopathological responses in a pandemic situation [3,23,24,25,26,27,28].

The number of coping strategies for lifestyle changes due to the pandemic and lockdown is significant, and they are mainly dependent on one’s personality traits. Besides, different coping styles are in close relationship with the severity of mental and emotional distress [29,30]. It is assumed that coping strategies might be predicted based on the combination of psychological factors, factors experienced during the pandemic and lockdown specifically, as well as demographic factors of the population [31]; they are also determined by gender, age, education, physical and mental health, and nature of the stressor [32,33]. Even though, during the pandemic, students most prevalently presented increased depression and/or anxiety and/or stress levels, they also tended to use approach coping strategies (such as acceptance, planning, and seeking emotional support) rather than avoidant coping strategies (such as denial, substance use, behavioral disengagement) [34,35].

The aim of this cross-sectional study that involves more than two thousand Polish students was to present the most frequently applied coping strategies amongst students during the ‘first wave’ of the COVID-19 pandemic. In addition, we aimed to present the effects of the pandemic on mental health, with an emphasis on emotional distress, as well as the severity of depression, anxiety, and stress symptoms. Lastly, we wanted to indicate adaptive coping strategies, which might be related to the reduction of emotional distress, acting as potential protective factors against the negative consequences of the pandemic, while at the same time presenting maladaptive coping strategies, leading to extremely high levels of anxiety, depression, and stress.

## 2. Materials and Methods

### 2.1. Study Design and Survey Description

Before designing the study and questionnaire, the authors conducted research on currently available literature regarding coping strategies for stress management amongst students during lockdown because of the COVID-19 pandemic. Then, an anonymous online survey questionnaire was created as a Google Forms document, which contained questions related to (1) sociodemographic data, (2) health status, and (3) economic situation, as well as Polish adaptations of the two scales—(4) the Depression, Anxiety, Stress Scale-21 (DASS-21) to assess emotional distress [36], (5) and the Carver Brief COPE Inventory (Brief-COPE) to assess coping strategies for stress in a population of Polish students [37]. The snowball sampling method was used, and the questionnaire was distributed among students via social media such as Facebook. We also sent e-mails to several universities with a request to send the questionnaire further. The questionnaire remained open from 20 to 26 April 2020 during the lockdown in Poland, which was officially announced by the government on 10 March 2020. The cross-sectional questionnaire was completed by students from 87 universities in Poland, including all medical universities. The questionnaire completion time was estimated at approximately 10 min. The obtained results were only used for scientific purposes and remained confidential for the researchers involved in this study.

The study was approved by the Bioethical Committee of the Medical University of Lublin, Lublin, Poland, and was conducted in compliance with national legislation and the Declaration of Helsinki. All of the respondents were asked to sign a consent form provided at the beginning of the questionnaire, stating that they were willing to take part in this study voluntarily. A consent form included a brief description of the study along with the information about confidentiality and anonymity; it also included information about the purpose, method, and personal data processing with regards to our online anonymous questionnaire. The respondents were informed that the results of this study were to be used only for scientific purposes and managed by the researchers involved in this study.

### 2.2. Measures

The questions selected for this cross-sectional questionnaire concerned the most crucial aspects of the respondents’ health status and current economic situation; further, the respondents were also asked to assess their emotional distress (measured by DASS-21) and indicate their coping strategies for stress during the COVID-19 pandemic (measured by the Brief-COPE).

The sociodemographic data referred to (1) sex, (2) age, (3) field of study, (4) year of study, (5) place of residence, (6) place of residence during the COVID-19 pandemic, (7) marital status, and (8) living situation and co-residence. Health status was assessed by asking questions related to (1) having a chronic disease, (2) mental problems, (3) using psychological or psychiatric services during and before the pandemic outbreak, (4) subjective difficulties experienced during lockdown, (5) taking immunity-boosting supplements, (6) being infected by the SARS-CoV-2, (7) COVID-19 cases among family/friends, and (8) deaths due to COVID-19 among family/friends. Economic status included issues that referred to (1) employment status during and before the pandemic outbreak and (2) assessment of the respondents’ economic situation during the pandemic.

### 2.3. Depression, Anxiety, and Stress Scale—21 Items (DASS-21)

The overall emotional distress, as well as the intensity of respondents’ depression, anxiety, and stress, were assessed by the Polish adaptation of the Depression, Anxiety, and Stress Scale—21 items (DASS-21) [36]. DASS-21 is a shortened version of the DASS-42 scale by Lovibond [38]; it was designed to measure the overall emotional distress, depression, anxiety, and stress levels by three separate scales composed of 14 items, and is further divided into several subscales. Each of the scales assesses different aspects: (1) depression scale assesses devaluation of life, dysphoria, inertia, anhedonia; (2) anxiety scale assesses autonomic arousal, situational anxiety, feelings of anxiety; while (3) stress scale assesses nervous arousal, relaxing difficulty, agitation. The respondents are asked to assess the above-mentioned items on a 4-point severity scale; DASS-21 assesses them in relation to respondents’ experiences over the past week. Three cutoffs—normal, moderate, and severe—are recommended while providing final DASS-21 scores. The total score of the DASS-21 ranges from 0 to 63, while for each of the subscales, it ranges from 0 to 21. The higher the score achieved, the greater the intensity of the depression, anxiety, and stress—the studied emotional disturbances. We have chosen to use this shortened version of the DASS-42 scale in order to reduce the questionnaire completion time to a minimum, while at the same time providing results of similar quality and consistency. Likewise, DASS-21 is much more frequently used for research, as opposed to clinical purposes [39]. An evaluation of the Polish version of the Depression, Anxiety and Stress Scale conducted on 212 medical students at Jagiellonian University Medical College in Kraków showed a very good internal consistency, with the Cronbach’s alpha value of DASS Total equal to 0.93, and for the three consecutive subscales: stress 0.85, anxiety 0.80, depression 0.86 for the Polish student population [40]. In our study, the alpha coefficients for the reliability of the depression, anxiety, stress, and full scale in the entire group were 0.95, 0.89, 0.96, and 0.94, respectively. The calculated values of Cronbach’s alpha for individual scales indicated the high reliability of the used scale [8].

### 2.4. The Carver Brief Cope Inventory (Brief-COPE)

The Carver Brief Cope Inventory (Brief-COPE) was used in our study to search for the most prevalent stress coping strategies amongst Polish students during the pandemic; it is a shortened version of the COPE inventory questionnaire designed by Carver et al. [37]. Brief-COPE is an abridged version of the 60-point COPE tool created by Carver, Scheier, and Weintraub. Both are self-reported scales that are used to measure which stress coping strategies are the most common stress coping strategies. In the presented study, the Polish adaptation of Brief-COPE was used. The Brief-COPE consists of 28 statements, which are the components of 14 major strategies for coping with stress (two statements for each strategy). For each statement, the respondent chooses one of four possibilities:

(1) *I almost never do this.*
(2) *I rarely do this.*
(3) *I do this often.*
(4) *I almost always do this.*

The test sheet is available on the website of the Psychological Test Laboratory at www.practest.com.pl; it is free to download and use for research purposes. There are many different interpretations of the Brief-COPE scale [41,42,43]. We decided to use the concept most commonly applied in the Polish adaptation of the Brief-COPE scale [37]. Approach coping strategies are portrayed by the following subscales: active coping, positive reframing, planning, acceptance, seeking emotional support, and seeking informational support. Avoidant coping strategies include denial, substance use, venting, behavioral disengagement, self-distraction, and self-blame. Humor and religion are neither avoidant nor approach coping strategies and form independent factors. The internal compliance of the Polish version of Mini-COPE was established based on a study of 200 people aged 25–60 years. The split-half reliability was 0.86. The constancy is satisfactory for most scales. The factor loadings of the individual theorems can mostly be regarded as satisfactory [37]. In our study, the Cronbach’s alpha coefficient was equal to 0.76.

## 3. Statistical Analysis

The statistical analysis applied in the following study included descriptive statistics, distribution of the numbers with a percentage distribution, H Kruskal–Wallis test, U Mann–Whiney test, multiple regression, r-Spearman correlation, and cluster analysis with dendrogram plot. Analysis of histograms and the Shapiro–Wilk test were used to check the distribution of the psychological variables. The distribution in particular scales and the overall result deviated (statistically significant) from the normal distribution, which means that most of the cases were not close to the mean. All calculations were conducted with Statistica v.13 program (Statistica software—Polish version from StatSoft Corporation Poland, the partner of Tibco Corporation, Palo Alto, CA, USA (license for the Medical University of Lublin)).

We standardized the scores of the DASS-21 total score to determine the risk factors for developing a higher intensity of emotional distress; afterward, we distinguished two groups of subjects for further comparisons: those with low and high DASS scores. The respondents with low scores (DASS 0; below mean (M)—standard deviation (SD)) constituted the first group of subjects, while those with high scores (above M + SD) were allocated to the second group.

## 4. Results

### 4.1. Sociodemographic Characteristics of the Respondents

The questionnaire was completed by 2172 students from 87 Polish universities, of whom 73% (*n* = 1.585) were women and 27% (*n* = 587) were men. The mean age of the respondents was 22.1 ± 2.2. The majority of the respondents were medical students (60.5%, *n* = 1314), and the remainder were students of the following fields of study: social sciences (19.2%, *n* = 416), engineering (10%, *n* = 219), arts and humanities (5%, *n* = 110), and sciences (4.4%, *n* = 96). The highest number of students were in their first year of study (23.5%, *n* = 511), and the lowest were completing their sixth year (4.9%, *n* = 106). The most common chosen place of residence was the countryside (22.9%, *n* = 497). The majority of the respondents were single (65.6%, *n* = 1.426) or in an informal relationship (30.7%, *n* = 667), while only 2.5% (*n* = 54) were married. Most of the students (48.3%, *n* = 1049) lived with their parents during the outbreak of the COVID-19 pandemic, while only a small percentage, that is, 1% (*n* = 21), lived with a partner and a child (Table 1).

### 4.2. Health Status of the Students Enrolled in the Study

According to the results of our questionnaire, the majority of students (97.2%, *n* = 2112) did not confirm that they were infected by SARS-CoV-2. In addition, 89.5% (*n* = 1943) replied that neither their relatives nor friends had had COVID-19. Only 1.3% (*n* = 28) of the students, 2.4% (*n* = 52) of their family members, and 7.4% (*n* = 161) of their friends reported that they were infected by the virus. Regarding psychological well-being, 82.9% (*n* = 1800) of the studied group used the support of neither a psychologist nor a psychiatrist before the outbreak of the pandemic, 7.5% (*n* = 162) used the help of a psychologist, and 3.7% (*n* = 80) visited a psychiatrist, while 6.0% (*n* = 130) used both—psychological and psychiatric support—even before the pandemic started. Taking into account somatic diseases, the majority of students (84.6%, *n* = 1837) did not report any chronic disease. Thyroid diseases (4.1%, *n* = 88), asthma (2.5%, *n* = 54), mental disorders (1.9%, *n* = 41), allergies (1.5%, *n* = 32), and diabetes (0.7 %, *n* = 15) were among the most frequently mentioned by the respondents. Of the total respondents, 69.3% (*n* = 1506) admitted that they did not use any dietary supplements during the pandemic. Those who wanted to boost their immune system (30.6%, *n* = 665) used a complex of vitamins (6.2%, *n* = 134), vitamin C (6.5%, *n* = 140), vitamin D (7.2%, *n* = 157), magnesium, omega-3 fatty acids, herbs, and traditional medicine (10.8%, *n* = 234) habitually. Due to the outbreak of the pandemic, students had to face a vast number of difficulties, the most pronounced being the fear of infection of loved ones (33.5%, *n* = 728), followed by the fear of changes that awaited the world after the pandemic (20.3%, *n* = 440), and change of lifestyle (12.2%, *n* = 265).

### 4.3. Economic Condition of Students Participating in the Study

Out of all the students, 67.6% (*n* = 1469) admitted that they did not work during the pandemic; only 30.9% (*n* = 672) worked either intellectually or physically. During the pandemic, 15.2% (*n* = 330) of the studied group lost their jobs. Furthermore, we asked respondents how they assessed their economic situation in this difficult time—more than half of them (58.8%, *n* = 1278) had a stable family income and nothing had changed, while 29.8% (*n* = 647) had a stable family income but described the situation as worse than before. Only 1% (*n* = 21) of the students answered that they had to start borrowing money from family or friends during the pandemic outbreak because they were not able to support themselves.

### 4.4. Total DASS Score

The DASS total score for the whole group of respondents was 38.13 ± 26.51, which is lower than the cut-off score equal to 60, as originally proposed by Lovibond and Lovibond [39]. Overall emotional distress turned out to be significantly higher (*p* < 0.001) in females (M = 40.54 ± 26.65) compared to males (M = 31.60 ± 25.02). There were statistical differences between the DASS total score and the field of study; the lowest DASS total score was observed in the case of medical students (median (Me) = 31.00 ± 25.48), while the highest was in the case of students studying science (Me = 42.00 ± 40.91), followed by arts and humanities (Me = 39.00 ± 28.98), social sciences (Me = 35.00 ± 28.14), and engineering (Me = 32.00 ± 26.79). Based on the Kruskal–Wallis test, the studied groups were statistically different (H = 16.16, *p* = 0.0028); the highest difference was noted between the medical and science students (z = 3.312, *p* = 0.009) (Supplementary Tables S1–S12).

### 4.5. Depression

The respondents presented a moderate level of depression symptoms; the mean score for the depression subscale for the entire group was equal to 14.04 ± 10.44. Depression symptoms were significantly (*p* < 0.001) more intensified amongst females (M = 18.41 ± 14.05) compared to males (M = 12.24 ± 14.37). The intensity of depression defined in the DASS-21 subscale concerned the following number of respondents: ‘normal’ depression applied to 43.6% (*n* = 948) of the students, ‘mild’ depression applied to 13% (*n* = 282), ‘moderate’ depression applied to 19.9% (*n* = 432), while ‘severe’ and ‘extremely severe’ applied to 10.2% (*n* = 221) and 13.3% (*n* = 289) of the students, respectively. A significant difference was found between the severity of depression for students of science and medicine (z = 3.25, *p* = 0.012), following the results obtained from the Kruskal–Wallis test (H = 19.82, *p* = 0.0005) (Supplementary Tables S2 and S55).

### 4.6. Anxiety

Regarding the anxiety subscale, the mean result for the entire group was 7.71 ± 8.29, which is equal to the ‘mild’ intensity of anxiety according to the DASS-21 scale. The intensity of anxiety was as follows in a sample of the studied students: ‘normal’ anxiety—60.2% (*n* = 1307), ‘mild’ anxiety—12.5% (*n* = 273), ‘moderate’ anxiety—9% (*n* = 195), ‘severe’ anxiety—6.6% (*n* = 144), and ‘extremely severe’—11.7% (*n* = 273). Similarly, in the depression subscale, females tended to present statistically higher (*p* < 0.001) anxiety levels (M = 13.19 ± 11.54) compared to males (M = 6.90 ± 9.92). Students studying arts and humanities (Me = 6.00 ± 8.63), science (Me = 6.00 ± 9.2), and social sciences (Me = 6.00 ± 8.53) reached the highest levels of anxiety, while in the case of medicine (Me = 4.00 ± 8.0) and engineering (Me = 4.00 ± 8.06), the intensity of anxiety was lower. However, the results did not vary significantly between the fields of studies (Supplementary Tables S3 and S56).

### 4.7. Stress

The mean score for the stress subscale was equal to 16.93 ± 10.98, which according to the DASS-21 scale can be classified as ‘mild’ stress. Similarly to the depression and anxiety subscales, females presented significantly (*p* < 0.001) higher levels of stress (M = 20.93 ± 15.45) compared to males (M = 12.58 ± 14.70). Stress levels amongst the respondents were of the following degrees of intensity: ‘normal’—47.2% (*n* = 1026), ‘mild’—11.8% (*n* = 255), ‘moderate’—15.3% (*n* = 333), ‘severe’—16.8% (*n* = 364), and ‘extremely severe’—8.9% (*n* = 194). Regarding stress levels, the highest stress intensity was observed in the case of the students studying science (Me = 20.00 ± 11.12), followed by art and humanities (Me = 19.00 ± 11.62), social sciences (Me = 16.00 ± 11.51), engineering (Me = 16.00 ± 10.82), and medicine (Me = 16.00 ± 10.73). There was no significant difference between stress intensity and the students’ field of study, based on the results obtained from the Kruskal–Wallis test (Supplementary Tables S4 and S57). The above results are presented in Table 2.

### 4.8. Coping Strategies in a General Population of Polish Students

Polish students chose stress coping strategies belonging to the ‘approach’ coping strategies more often (M = 29.60 ± 6.89) compared to ‘avoidant’ coping strategies (M = 22.82 ± 5.78). Amongst the most frequently chosen stress coping strategies were acceptance (M = 2.19 ± 0.71), emotional support (M = 1.58 ± 0.93), planning (M = 1.44 ± 0.84), and positive reframing (M = 1.41 ± 0.90), as well as one strategy belonging to the ‘avoidant’ group, namely self-distraction (M = 1.43 ± 0.80). Further, the three most rarely chosen strategies were denial (M = 0.43 ± 0.64), substance use (M = 0.50 ± 0.80), and behavioral disengagement (M = 0.70 ± 0.76), which also belong to avoidant coping (Supplementary Tables S13 and S14).

### 4.9. Sex Differences in the Choice of Coping Strategies between Females and Males

The most frequently chosen stress coping strategy by females was acceptance (M = 2.18 ± 0.71), followed by emotional support (M = 1.67 ± 0.92) and planning (M = 1.46 ± 0.84), while in the case of males, the most prevalent ones also included acceptance (M = 2.22 ± 0.70) and planning (M = 1.38 ± 0.87), as well as humor (M = 1.33 ± 0.74).

Mann–Whitney’s U test showed that among the avoidant coping strategies, women differed from men, choosing self-distraction (M = 1.51 ± 0.79, *p* < 0.001), denial (M = 0.47 ± 0.66, *p* < 0.001), and venting (M = 1.37 ± 0.76, *p* = 0.0001) more often, while men resorted to substance use (M = 0.61 ± 0.85, *p* < 0.001). Among the approach strategies, more women chose emotional support (M = 1.67 ± 0.92, *p* < 0.001), use of informational support (M = 1.27 ± 0.87, *p* < 0.001), and positive reframing (M = 1.45 ± 0.89, *p* = 0.001). While women turned to religion (M = 0.84 ± 1.00, *p* = 0.001), men dealt with the situation through a sense of humor (M = 1.33 ± 0.74, *p* < 0.001) (Table 3).

### 4.10. Stress Coping Strategies and Fields of Study

The respondents’ fields of study included medicine, art and humanities, social sciences, engineering, and sciences. The analysis showed a statistically significant differences in the gender distribution of the respondents in the analyzed fields of study (Chi-kw = 109.22, df = 4, *p* = 0.00001), which justifies conducting analyses with division into fields of study. Significant differences were noted between medical and social sciences students, which resulted only from differences between women in terms of preferred strategies (Supplementary Tables S58–S65). Out of 14 Brief-COPE strategies, in five of them (i.e., ‘active coping’, ‘use of informational support’, ‘planning’, ‘humor’, and ‘self-blame’), female medical students achieved significantly higher results than female social sciences students. This is an interesting observation, especially in the context of the lack of any differences between males in this regard.

Students from all fields of study most frequently chose ‘acceptance’ as a coping strategy for stress, except for the engineering students, who most often used ‘positive reframing’. Other frequently chosen coping strategies included emotional support, planning, positive reframing, and self-distraction. Amongst students studying sciences, ‘planning’ was most prevalently replaced by ‘venting’ instead. Based on the results of the Kruskal–Wallis test, students of particular fields of study differed statistically in their chosen coping strategies. Regarding the use of informational support, the individual fields of study were statistically different (H = 20.74, *p* = 0.0004), with medicine and engineering presenting the most pronounced difference (z = 3.70, *p* = 0.002). The use of the venting strategy also differed significantly between students of different fields of study (H = 18.39, *p* = 0.0010); the highest difference was noted between medicine and engineering (z = 3.83, *p* = 0.001), followed by sciences and engineering (z = 3.35, *p* = 0.008). Planning also showed a statistical difference (H = 25.30, *p* < 0.0001), namely between medicine and social sciences (z = 4.16, *p* < 0.001). The groups also differed statistically in terms of sense of humor in stressful situations (H = 22.39, *p* = 0.0002). The most significant difference was observed between medicine and social sciences (z = 4.38, *p* < 0.001), followed by engineering and social sciences (z = 3.40, *p* = 0.007). Acceptance showed the biggest difference (H = 37.08, *p* < 0.0001) between medical students and social sciences students (z = 5.45, *p* = 0.000). The turn to religion also showed a statistical difference (H = 27.07, *p* < 0.0001) between the studied groups, particularly in the case of medicine and social sciences (z = 3.95, *p* = 0.001), (Supplementary Tables S15–S19 and S47–S52).

### 4.11. Coping Strategies of Students who Were Using Support of a Psychologist or Psychiatrist before a Pandemic

The majority of respondents (*n* = 1800) used the support of neither a psychologist nor a psychiatrist before the outbreak of the pandemic. The study showed that the three most popular strategies for coping with stress belonged to the approach category and included acceptance (M = 2.19 ± 0.71), emotional support (M = 1.57 ± 0.92), and planning (M = 1.42 ± 0.84). Interestingly, the coping strategies of students who used the support of a psychologist or psychiatrist before the pandemic presented in the same order. Other results were achieved by students who had to use the support of both a psychologist and a psychiatrist—the most common coping strategies in this group were acceptance (M = 2.17 ± 0.71), emotional support (M = 1.52 ± 0.99), and self-distraction (M = 1.52 ± 0.83), (Supplementary Tables S20–S23).

### 4.12. Coping Strategies of Students Depending on the Living Situation

Regardless of who the respondents lived with, acceptance was always the most common coping strategy in the cases of living alone (M = 2.26 ± 0.77), with roommates (M = 2.21 ± 0.67), with parents (M = 2.16 ± 0.72), with a partner or spouse (M = 2.24 ± 0.70), and with a partner or spouse and a child (M = 2.12 ± 0.71), (Supplementary Tables S24–S28).

### 4.13. The Most Prevalent Difficulties during the Pandemic and Associated Coping Strategies

Regardless of the type of situation that respondents perceived as the most difficult, the most common strategy were acceptance. One of the greatest difficulties pointed out by the students was fear of the infection of their loved ones (*n* = 730). The three most frequently chosen strategies for coping with stress in this situation included acceptance (M = 2.21 ± 0.67) and emotional support (M = 1.70 ± 0.90), as well as one avoidant coping strategy—self-distraction (M = 1.49 ± 0.80). Quantitatively, the second most frequently chosen difficulty was fear of the changes that awaited the world after the pandemic (*n* = 446). Those students most often used approach coping strategies: acceptance (M = 2.28 ± 0.68), emotional support (M = 1.66 ± 0.92), and planning (M = 1.59 ± 0.87). The lowest number of people (*n* = 26) chose the answer that they were not afraid of anything; the strategies most often used by this group were acceptance (M = 2.40 ± 0.84), humor (M = 1.85 ± 0.96), and positive reframing (M = 1.21 ± 1.12).

The use of the approach coping strategy was statistically different (H = 43.75, *p* = 0.00001) between those students who felt fear of infection of their loved ones and for those who struggled to cope with loneliness (z = 3.92, *p* = 0.003). Another statistical difference was shown by those who felt loneliness the most, and those who were afraid of the changes in a post-pandemic world (avoiding repetition) (z = 3.88, *p* = 0.004). Another difference concerned those who were not afraid of anything, and those who were also afraid of the uncertainty of an altered world (z = 3.84, *p* = 0.004). Statistical differences were also shown in the use of an avoidant coping strategy by students in the context of the greatest difficulties experienced during lockdown (H = 79.18, *p* < 0.0001). The greatest difference was shown between loneliness and those students who were not afraid of anything (z = 7.29, *p* < 0.001), followed by the fear of being infected and those who felt no fear (z = 5.91, *p* < 0.001), and additionally, between those who had a concern about their education and those who felt lonely (z = 5.48, *p* < 0.001), (Supplementary Tables S29–S37, S53 and S54).

### 4.14. Coping Strategies of Students Depending on Their Employment Status

Despite their occupation, all students who were not working (M = 2.19 ± 0.70), were working intellectually (M = 2.20 ± 0.73), physically (M = 2.16 ± 0.76), or ran their own business (M = 2.34 ± 0.77) chose acceptance as a major stress coping strategy. Except for acceptance, in all of the aforementioned groups, students most prevalently chose emotional support, planning, self-distraction, and positive reframing (Supplementary Tables S38–S46).

### 4.15. Coping Strategies of Students Suffering from Chronic Diseases

Overall acceptance was the most frequently chosen stress coping strategy for students without chronic disease (*n* = 1837), as well as those with thyroid disease (*n* = 88), asthma (*n* = 54), allergy (*n* = 32), diabetes (*n* = 15), or mental disorders (*n* = 41), as well as students with other chronic diseases (*n* = 105), including gastroenterological, neurological, circulatory, endocrine, or autoimmune diseases. The differences between the groups of students suffering from various chronic diseases were related to less frequently used strategies. The second most commonly used strategy for all groups was emotional support, except for asthmatics, who chose planning (M = 1.54 ± 0.82), and those suffering from mental disorders, who used an avoidant strategy of coping with stress—self-distraction (M = 1.78 ± 0.76). Additionally, students suffering from mental disorders most often chose negative ways of coping with stress—the third most prevalently chosen strategy was venting (M = 1.67 ± 0.85). Among the 14 strategies, students of all groups chose substance use and denial least frequently, apart from those suffering from mental disorders, who were least likely to choose religion as a way of coping (M = 0.60 ± 0.87) (Supplementary Tables S5–S11).

### 4.16. Correlation between Stress Coping Strategies and Level of Emotional Distress

A strong positive relationship has been observed between avoidant coping mechanisms and scores of DASS Total (r = 0.649), depression (r = 0.588), anxiety (r = 0.556), and stress (r = 0.591). Conversely, approach coping mechanisms show a weak and very weak negative correlation with DASS Total (r = −0.068) and depression (r = −0.159), respectively, both of which were statistically significant (Table 4).

According to the results and statistical significance, the highest positive correlation was found between the scores of DASS Total and the level of depression (D), anxiety (A), and stress (S), and the same three coping mechanisms: self-blame (DASS Total: r = 0.609; D: r = 0.596; A: r = 0.490; S: r = 0.537), behavioral disengagement (DASS Total: r = 0.603; D: r = 0.627; A: r = 0.466; S: r = 0.507), and venting (DASS Total: r = 0.457; D: r = 0.355; A: r = 0.397; S: r = 0.468). As for the highest negative correlation, it was found between acceptance (DASS Total: r = −0.257; D: r = −0.248; A: r = −0.212; S: r = −0.229) and positive reframing (DASS Total: r = −0.159; D: r = −0.201; A: r = −0.057; S: r = −0.141) in all DASS components. On the other hand, for anxiety levels, there were only two coping strategies shown above that were statistically important enough to have negative correlation. For people who used emotional support during the pandemic, there was a decreased level of depression (r = −0.175) and DASS Total scores (r = −0.088). Humor was found to have the third highest negative correlation with level of stress (r = −0.078). Furthermore, religion as a mechanism of stress coping, in addition to a very weak negative correlation with depression (r = −0.093), shows a very weak positive correlation with anxiety (r = 0.057).

### 4.17. The Impact of the Stress Coping Strategies Used by Polish Students on the Emotional Well-Being

According to the results of the multiple regression analysis, the results obtained in the following Brief-COPE scales have a significant impact (R = 0.760, R^2^ = 0.578 corrected R^2^ = 0.5759; F (14.2157) = 211.64 *p* < 0.00001, SE of E: 17.265) on the increase of the total DASS score: behavioral disengagement (*p* < 0.0001), venting (*p* < 0.0001), self-blame (*p* < 0.0001), and planning (*p* = 0.007); all of them belong to avoidant coping strategies, except for planning, which is an approach coping strategy (Table 5).

**Figure 1:**
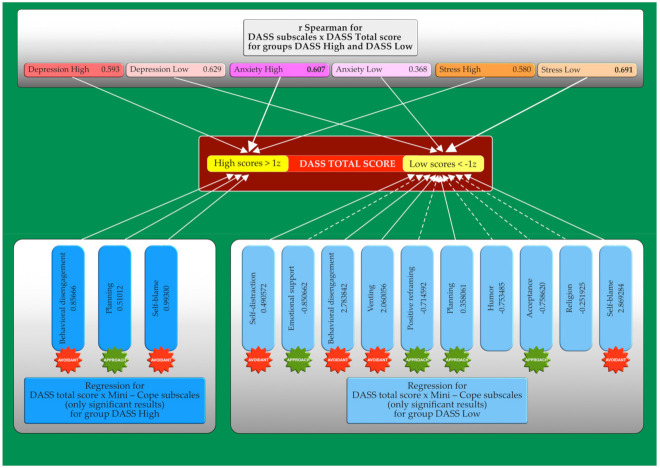
The impact of the stress coping strategies and DASS subscales on the emotional well-being (DASS Total score) of Polish students.

Contrarily, the following Brief-COPE scales have had a statistically significant impact on lowering the total DASS score: emotional support (*p* < 0.001), positive reframing (*p* < 0.001), acceptance (*p* < 0.001), and humor (*p* < 0.001). All of these scales belong to the approach coping strategies, except ‘humor’, which is an independent factor. The subscales of anxiety (r = 0.607), depression (r = 0.593), and stress (r = 0.580) correlated with higher DASS total scores, respectively. On the other hand, the stress subscale (r = 0.691) correlated with lower values of the DASS Total score, followed by depression (r = 0.629) and anxiety (r = 0.368) (Figure 1).

## 5. Discussion

Research devoted to the mental health of students in the COVID-19 era is currently ongoing all over the world. The uncertainty of returning to schools and universities, as well as changing the forms of teaching and isolation, significantly affected young adults. There are many examples indicating how the mental health of students deteriorated during the pandemic; one of them is a study of more than two thousand American college students, in which 48.14% (*n* = 960) showed a moderate-to-severe level of depression, 38.48% (*n* = 775) showed a moderate-to-severe level of anxiety, and 18.04% (*n* = 366) had suicidal thoughts [44]. The shift to online learning also had a significant impact on the mental health of students. Research shows that depression and stress levels increased due to e-learning [45]. A study involving 69,054 students living in France during the COVID-19 quarantine showed a high prevalence of mental health issues among students who experienced quarantine, underlining the need to reinforce prevention, surveillance, and access to care [46].

In this study, we examined how Polish students manage emotional distress during the COVID-19 pandemic. In our study, students preferred to choose ‘approach’ coping strategies (M = 29.60 ± 6.89), rather than ‘avoidant’ coping strategies for stress (M = 22.82 ± 5.78). Such data indicates that most of the students aimed to approach the problem of the ongoing COVID-19 pandemic in an adaptive way. The implementation of such coping strategies for stress was associated with lower emotional distress as well as lower depression, anxiety, and stress symptoms amongst Polish students. Furthermore, the intensification of distress in women causes a turn to religion (*p* = 0.001), while men use substances (*p* < 0.001) and a sense of humor (*p* < 0.001). The importance of religious practices for the mental condition of adults during the lockdown period in Poland is also confirmed by the results of other authors, especially in relation to private practice [47]. Acceptance was one of the most frequently chosen coping strategies regardless of gender, age, economic status, or health status of the respondents, followed by emotional support, planning, self-distraction, and positive reframing.

Considering the field of study, it turned out that medical and social sciences students differ in the chosen stress coping strategies. Further analysis showed that these differences are due only to differences between women studying medicine and social sciences. In five of the included Brief-COPE strategies (i.e., ‘active coping’, ‘use of informational support’, ‘planning’, ‘humor’, and ‘self-blame’), female medical students achieved significantly higher results than female social sciences students.

Regarding the results obtained using the DASS-21 scale, the total DASS score for the entire group of the respondents was 38.13 ± 26.51, while the subscales including depression, anxiety, and stress were equal to 14.04 ± 10.44, 7.71 ± 8.29, and 16.93 ± 10.98, respectively. Such results represent a moderate level of depression and mild stress and anxiety. Regarding the DASS Total score, it is lower than the cut-off score proposed by Lovibond and Lovibond [38]. Le Vigouroux et al. has also indicated that anxiety and depressive symptoms are highlighted amongst French students, but those variables cannot be compared exactly between our studies due to different psychological tools used by their research group (Hospital Anxiety and Depression Scale (HADS)). Regardless, it should be pointed out that similarly to Polish students, emotional distress was also triggered amongst French students during the COVID-19 outbreak. Similarly, in the case of Egyptian students, depression and stress symptoms were mostly enhanced during the outbreak of the pandemic [48].

Our results seem to be in agreement with those obtained by researchers from other countries, which were presented particularly as enhanced anger, insomnia, depression, overall exhaustion, or loss of willingness to conduct everyday tasks. Spanish students presented moderate to extremely severe symptoms of stress (28.14%), anxiety (21.34%), and depression (34.19%) [49]. The results of one of the Arabic studies indicated that a vast majority of students (92.9%) suffered from the elevation of psychological distress specifically during the outbreak of the COVID-19 pandemic [50]. It should be noted that there were gender differences regarding the intensity of the emotional distress, which was mostly intensified in females, indicating that they might be more vulnerable to depression, anxiety, and stress symptoms compared to males.

While searching PubMed, Scopus, and Web of Science databases, we found four similar studies conducted in Egypt, France, Nepal, and Pakistan, which we found interesting to compare due to the similarities in materials (population group) and methods used in our studies, along with the time when our studies were conducted [48,51,52,53]. Comparing the four most frequently chosen stress coping strategies between Polish, French, Egyptian, Nepalese, and Pakistani University students, we can see similarities. In all those countries, acceptance was the most popular strategy, except in Pakistan, where it was in second place and religious/spiritual coping was number one. Only one strategy belonging to the avoidant group was present in the top four strategies in almost all countries (except Egypt), in third or fourth position. Positive reframing was found in three countries, as well as planning, while active coping and religion were found in two countries. Only Polish students selected emotional support as the second most common strategy. Egyptian and Pakistani students were the only ones that had used religious coping mechanisms as one of the most common strategies. These dissimilarities could be due to cultural differences. The main religion of Egypt and Pakistan’s population is Islam, in comparison to Poland and France, where it is mostly Christianity, and in Nepal, where it is Buddhism [54]. The four most frequently chosen stress coping strategies are shown in Table 6.

Approach coping strategies were the most common strategies chosen by the students from Poland and were associated with better well-being. The strategies that played a generally protective role against emotional distress symptoms were emotional support, positive reframing, acceptance, and humor, as seen in Table 4. All other countries have a similar pattern, and show the same effect of approach strategies on the well-being of participants. Interestingly, self-distraction was among the four most frequently chosen coping strategies in all compared countries. In France, acceptance and positive reframing strategies played a generally protective role against anxiety and depressive symptoms. French students who most willingly chose avoidant coping strategies included those who were worried about their future job prospects; those who were worried about their own and their families’/friends’ health statuses; and those whose strategies mostly included behavioral disengagement or denial [51].

Concerning gender differences in Polish students, women more often chose emotional support (*p* < 0.001), use of informational support (*p* < 0.001), positive reframing (*p* = 0.001), self-distraction (*p* < 0.001), denial (*p* < 0.001), venting (*p* = 0.0001), and religion (*p* = 0.001), while men resorted to substance use (*p* < 0.001) and humor (*p* < 0.001). Significant differences were also observed in Pakistani students—gender was one of the major factors where significant differences were observed regarding the following coping strategies: planning, humor, acceptance, self-distraction, and religious coping. Specifically, females had significantly lower scores for planning (*p* = 0.033), as well as humor (*p* < 0.001), while males had significantly lower scores for acceptance (*p* = 0.019), religious coping (*p* < 0.001), and self-distraction (*p* < 0.001) [53]. In a study performed by Skapinakis et al., Greek students mostly used acceptance, humor, and planning as their major coping strategies during the pandemic outbreak, including both females and males, once again indicating that approach coping strategies were more preferable to the students [55]. Furthermore, the results of this study indicate that active/positive coping strategies were more frequently used compared to supportive strategies. Interestingly, more supportive coping strategies along with religious coping were more likely used by females, while males tended to apply substance use. In another study conducted by Salman et al., the most frequently chosen coping strategies included religious coping, acceptance, and planning, while the lowest prevalence was substance use and self-blame [53]. Regarding gender differences in this study, females were observed to have significantly greater scores for venting (*p* = 0.015), religious/spiritual coping (*p* = 0.003), and behavioral disengagement (*p* = 0.043), compared to males.

According to current research, the gender differences in coping strategies among medical students appear to be very diverse. For example, Dodek et al. showed that gender does not matter among medical students when choosing a strategy for coping with distress [56]. This observation is also confirmed by studies conducted on medical students from Canada and Scotland long before the outbreak of the pandemic [57,58]. However, some authors, such as Shaikh et al. found significant gender differences in coping. In Pakistan, women studied and slept, while men preferred to play sports, isolate themselves, or spend time with friends [59]. Additionally, in Pakistan, some studies have shown that it was men who more frequently displayed maladaptive coping strategies, such as substance use, denial, and self-blame [60]. Since all these observations concern both Western and Eastern countries, further research, especially in an exceptional situation such as increased distress due to pandemic, seems interesting, especially since cultural and environmental factors certainly also play a role.

Regarding gender differences, in a COVID-19 situation, a May 2021 study by Neufeld et al. showed that females reported greater use of behavioral disengagement, while men reported less trust in emotional and instrumental support [61]. The literature suggests that differences in coping with stress may be due to playing gender roles [62]. Thus, the study findings that females used more behavioral disengagement while men used less social support may reflect certain stereotypes. Perhaps men will not reach for social support because such behavior is antagonistic to traditional male ideals. On the other hand, women may choose behavioral disengagement more often to show traditional feminine traits such as being submissive. Considering the cultural factor, it is interesting that, as in our study, in South-West Nigeria and Malaysia, women turned to religion more often than men, which may also be associated with the cultivation of stereotypically adopted gender roles [63,64].

In addition, we observed a very interesting phenomenon by studying the literature and comparing the obtained results in terms of the study field. In Poland, medical students coped best with the pandemic, which is a very promising factor in the context of their future professional work. In Pakistan, medical students had significantly lower “self-blame” scores than pharmacy (*p* = 0.005), allied health sciences (*p* = 0.028), and other university students (*p* = 0.002) [53]. This showed quite a different approach than in Egypt, where medical students chose the most dysfunctional coping strategies compared to colleagues studying engineering, sciences, and humanities, and reached the highest levels of depression, anxiety, and stress [48]. The results of our study showed a relationship between the field of study and the DASS Total score. The highest intensity of emotional distress was obtained by science students, followed by those who studied arts and humanities, and then social sciences and engineering. Medical students also scored the lowest in the DASS Total score (Supplementary Table S1).

Searching for factors having the greatest impact on the emotional state of students during the lockdown, isolation and loneliness played a meaningful role. In our study, students who chose “loneliness” as their greatest difficulty during the pandemic were 292 times more likely to experience higher overall emotional stress [8]. Loneliness is a well-known factor in the development of depression, which can be further intensified by the loss of the ability to use natural coping techniques in a pandemic, which Dawson and Golijani-Moghaddam showed in their research [65,66]. In a study conducted in France by Vigouroux et al., it was argued that students found it tough to implement a variety of coping strategies during the lockdown. Even so, they were able to use several effective strategies such as acceptance, planning, and positive reframing. It is possible that students who were not flexible in their choices also showed the highest levels of anxiety and depression symptoms [51].

Reflecting on the intensity of emotional distress between students of different years of study, in Poland there were no statistical differences between the first-year students and the rest of the respondents in the DASS Total score, nor in any of the DASS subscales. The situation was different in Nepal, where last-year nursing students were the most depressed, stressed, and afraid compared to those in their first, second, or third year [52]. Nepalese colleagues suggest that it may be related to uncertainty about graduation and their professional future.

Even if the model of stress and coping by Lazarus and Folkman mentioned in our study is somewhat simplified (e.g., due to limitations related to internet research and the nature of brief tools), it indicates a linkage between stress, coping with stress, and basic emotions.

The results of our study can provide an insight into which of the coping strategies could act protectively for students and might possibly alleviate depression, anxiety, and stress symptoms effectively in the era of the COVID-19 pandemic. It is very encouraging to see such results since they indicate that students tend to be rather problem-focused and primarily chose positive coping strategies, indicating that they could probably adapt more easily to the changes associated with the ongoing pandemic. Further, such coping with the pandemic might lead to lower-intensity depression, anxiety, and stress symptoms compared to situations when students would rather use negative coping strategies.

The results of the study also have clinical implications for both preventive and therapeutic interventions, related to: (1) the urgent need to identify students particularly at risk of significant emotional disturbances during the COVID-19 pandemic; (2) designing therapeutic programs for the identified group of distressed students in need, including techniques to change maladaptive to effective and adaptive stress coping strategies; (3) creation of crisis/mental health help centers for students to cope with emotional distress caused by the pandemic; and (4) the necessity of including stress coping training in the curriculum of various fields of study.

## 6. Limitations of the Study

One of the limitations of this study was that the respondents were only Polish students; therefore, the obtained responses were limited to a very select group of students representing quite narrow cultural, ethnic, and religious groups. In Poland, Christianity is the most prevalently practiced religion, which could eventually affect the responses of the students - particularly their coping strategies—which is also emphasized in our results. Besides, the majority of the respondents (*n* = 1585) were females, while the field of study was also primarily limited to medical students (*n* = 1314). The students who were involved in this study included only those who were interested in this topic and wanted to contribute to this project. Therefore, our results cannot be generalized to other cultural, ethnic, or religious groups, and only represent those who were included in this study. In the discussion, we tried to compare our results with those obtained in other countries; however, cultural, ethnic, and religious differences, as well as varying restrictions and lockdown status should be taken into consideration, limiting the possibility of providing a fully reliable comparison.

As shown in our study, students of different fields of studies presented different intensities of overall emotional distress, along with the exacerbation of depression, anxiety, and stress symptoms, which, in some aspects, are associated with the application of various coping strategies. Our data is slightly limited since mostly medical students were the respondents, and they presented significantly lower levels of overall emotional distress compared to students in other fields of studies.

Moreover, the reliability of this study is slightly limited due to the fact that it was an online questionnaire, and we did not have any control over the respondents’ responses. Besides, since the questionnaire was open only from 20 to 26 April 2020, the results reflect only a short period of time, without any insight into the possible changes that could occur because of the implementation of further restrictions by the government. Even though we aimed to show the application of which coping strategies are associated with the lowest overall emotional distress and depression, anxiety, and stress levels, we cannot conclude whether their application will provide long-term results.

Another limitation was the choice of an online questionnaire type, which did not provide us information on what percent of students withdrew from the study and at what stage. This data would give us information about the respondents and the structure of the survey itself. Nevertheless, the conducted study provided great insight into the mental condition of the students, who constituted a group capable of going through the entire questionnaire. Besides, the study was conducted during the ‘first wave’ of the pandemic, and thus coping mechanisms may have changed dynamically to this day. Future studies can certainly give an insight into this issue.

## 7. Conclusions

The results of our study indicate that social isolation in the form of the lockdowns announced by governments worldwide, along with fear of the pandemic itself (associated with the limited knowledge about the virus and infection) might significantly contribute to the increase of emotional distress in the form of enhanced depression, anxiety, and stress symptoms. Summarily, the respondents showed different coping strategies for stress during the ongoing COVID-19 pandemic. However, even though coping strategies differed slightly amongst the respondents, the vast majority of them were approach coping strategies, rather than avoidant. A similar pattern was observed in other countries as well. This suggests that students were rather willing to cope positively with the pandemic. Nonetheless, it is worth noting that emotional distress was also triggered amongst students, thus indicating that students constitute a group that should be monitored in terms of their mental health since they are also the most prone to be affected by changes associated with the ongoing pandemic. Psychological interventions are essential to address the mental health problems among university students during current and future disasters.

The results obtained in the study show that maladaptive coping strategies, especially in the prevailing pandemic, may consequently lead to unfavorable results of psychophysiological health, and thus the education and professional development of students. Referring to such observations and the model of co-participation in undertaking interventions, creation of a program that includes not only psychological but also instrumental and institutional support is highly desirable and recommended.

## Figures and Tables

**Table 1:** Sociodemographic characteristics of 2172 respondents included in the study.

Question	Answer	Number of Respondents	% of Respondents
**Mean age**	22.15	2172	100
**Sex**	Women	1585	73.0
	Men	587	27.0
**Field of study**	Arts and humanities	110	5.0
	Sciences	96	4.4
	Medicine	1314	60.5
	Engineering	219	10.0
	Social sciences	416	19.2
**Year of study**	I	511	23.5
	II	444	20.4
	III	507	23.3
	IV	322	14.8
	V	277	12.8
	VI	106	4.9
**Place of residence** **(number of inhabitants)** **[in thousands]**	Village	497	22.9
	Less than 20	219	10.1
	20–100	344	15.8
	100–300	276	12.7
	300–600	409	18.8
	More than 600	427	19.7
**Marital status**	Single	1426	65.6
	Informal relationship	667	30.7
	Married	54	2.5
**Do you have children?**	No	2130	98.1
	One child	20	0.9
**I live with:**	Alone	231	10.6
	Parents	1049	48.3
	Roommates	565	26.0
	Partner or spouse	301	13.9
	Partner/spouse and children	21	1.0

**Table 2:** Results of depression, anxiety, and stress severity for the whole group based on the DASS-21 scale.

DASS	N	Mean	SD
**DASS Total score**	2172	38.13	26.51
**Depression**		14.04	10.44
**Anxiety**		7.71	8.29
**Stress**		16.93	10.98

**Table 3:** The results of the most frequently chosen stress coping strategies for each gender.

Coping Stress Strategy	Women			Men			U Mann–Whitney Test
	Mean	Median	SD	Mean	Median	SD	*p*
Self-distraction	1.51	1.50	0.79	1.19	1.00	0.79	0.0001
Active coping	1.02	1.00	0.79	0.96	1.00	0.81	0.119
Denial	0.47	0.00	0.66	0.31	0.00	0.59	0.0001
Substance use	0.46	0.00	0.77	0.61	0.00	0.85	0.0001
Emotional support	1.67	2.00	0.92	1.31	1.50	0.88	0.0001
Use of informational support	1.27	1.00	0.87	0.94	1.00	0.79	0.0001
Behavioral disengagement	0.72	0.50	0.77	0.65	0.50	0.73	0.112
Venting	1.37	1.50	0.76	1.04	1.00	0.75	0.0001
Positive reframing	1.45	1.50	0.89	1.30	1.50	0.90	0.001
Planning	1.46	1.50	0.84	1.38	1.50	0.87	0.059
Humor	1.00	1.00	0.67	1.33	1.50	0.74	0.0001
Acceptance	2.18	2.50	0.71	2.22	2.50	0.70	0.279
Religion	0.84	0.50	1.00	0.65	0.00	0.87	0.001
Self-blame	0.76	0.50	0.69	0.73	0.50	0.72	0.218

**Table 4:** Correlation analysis results of COPE subscales, DASS subscales, and total score.

Variable	Depression	Anxiety	Stress	DASS Total Score
Self-distraction, items 1 and 19 (avoidant)	0.201 *	0.283 *	0.297 *	0.291 *
Active coping, items 2 and 7 (approach)	−0.011	0.105 *	0.083 *	0.064 *
Denial, items 3 and 8 (avoidant)	0.296 *	0.338 *	0.292 *	0.335 *
Substance use, items 4 and 11 (avoidant)	0.215 *	0.219 *	0.199 *	0.234 *
Emotional support, items 5 and 15 (approach)	−0.175 *	−0.024	−0.025	−0.088 *
Use of informational support, items 10 and 23 (approach)	0.004	0.130 *	0.134 *	0.097 *
Behavioral disengagement, items 6 and 16 (avoidant)	0.627 *	0.466 *	0.507 *	0.603 *
Venting, items 9 and 21 (avoidant)	0.355 *	0.397 *	0.468 *	0.457 *
Positive reframing, items 12 and 17 (approach)	−0.201 *	−0.057 *	−0.141 *	−0.159 *
Planning, items 14 and 25 (approach)	−0.004	0.109 *	0.101 *	0.071 *
Humor, items 18 and 28	−0.005	−0.030	−0.078 *	−0.041 *
Acceptance, items 20 and 24 (approach)	−0.248 *	−0.212 *	−0.229 *	−0.257 *
Religion, items 22 and 27	−0.093 *	0.057 *	−0.011	−0.024
Self-blame, items 13 and 26 (avoidant)	0.596 *	0.490 *	0.537 *	0.609 *

Coefficients marked with * are significant with *p* less than 0.05. Green—approaches with three highest-strength negative correlations, red—approaches with three highest-strength positive correlations.

**Table 5:** Summary of multiple regression analysis (N = 2157).

Variable	B	SE (B)	β	*t*	Sig. (*p*)
Constants			25.234	16.700	0.0000001
**Self-distraction**	0.037	0.020	1.243	1.847	0.064
**Active coping**	−0.026	0.017	−0.893	−1.522	0.127
**Denial**	0.030	0.016	1.245	1.843	0.065
**Substance use**	0.028	0.015	0.933	1.822	0.068
**Emotional support**	−0.116	0.020	−3.343	−5.663	0.0000001
**Use of informational support**	0.038	0.021	1.175	1.751	0.079
**Behavioral disengagement**	0.322	0.018	11.306	17.837	0.0000001
**Venting**	0.236	0.021	8.123	11.095	0.0000001
**Positive reframing**	−0.094	0.016	−2.799	−5.589	0.0000001
**Planning**	0.049	0.018	1.555	2.685	0.007
**Humor**	−0.079	0.015	−2.986	−5.276	0.0000001
**Acceptance**	−0.076	0.016	−2.861	−4.572	0.000005
**Religion**	−0.037	0.015	−1.027	−2.499	0.012
**Self-blame**	0.304	0.017	11.509	17.184	0.0000001

Note: R^2^ = 0.578; F (14.2157) = 211.64; *p* < 0.00001.

**Table 6:** Comparison between studies regarding most frequently chosen coping strategies.

Country	Poland		Egypt [48]	France [51]	Nepal [52]	Pakistan [53]
**Date of the start and closure of the survey**	20 April–26 April		30 May–6 June	23 April–8 May	13 June–10 July	11 April–24 April
**The time from confinement until the start of the survey**	6 weeks		9 weeks	4 weeks	12 weeks	3 weeks
**Number of the respondents**	2172		612	1297	207	1134
**Females** **(%)**	73		61.8	77.79	ND	70.5
**Males** **(%)**	27		38.2	20.66	ND	29.5
**Mean age/age range**	22.1 ± 2.2		ND	21.27 ± 4.72	ND	21.7 ± 3.5
The four most frequently chosen strategies	**1**	acceptance	acceptance	acceptance	acceptance	religious/spiritual coping
	**2**	emotional support	religion	positive reframing	positive reframing	acceptance
	**3**	planning	planning	planning	self-distraction	self-distraction
	**4**	self-distraction	positive reframing	self-distraction	active coping	active coping

Green—approach coping strategies, red—avoidant coping strategies, blue—independent coping strategies, ND—no data.

## Data Availability

The data presented in this study are available on request from the corresponding author.

## Supplementary Materials

The following are available online at https://www.mdpi.com/2077-0383/10/21/4964/s1, Table S1. The results of the DASS Total score for each field of study, Table S2. The results of the DASS depression scale for each field of study, Table S3. The results of the DASS anxiety scale for the field of study, Table S4. The results of the DASS stress scale for the field of study, Table S5. The results of the most frequently chosen stress coping strategies for having other chronic disease, Table S6. The results of the most frequently chosen stress coping strategies without having a chronic disease, Table S7. The results of the most frequently chosen stress coping strategies for thyroid diseases, Table S8. The results of the most frequently chosen stress coping strategies for asthma, Table S9. The results of the most frequently chosen stress coping strategies for allergy, Table S10. The results of the most frequently chosen stress coping strategies for diabetes, Table S11. The results of the most frequently chosen stress coping strategies for mental disorders, Table S12. The results of the DASS total score for females and males, Table S13. The results of the most frequently chosen type of coping strategies by all respondents, Table S14. The results of the most frequently chosen stress coping strategies by all respondents, Table S15. The results of the most frequently chosen stress coping strategies by medical students, Table S16. The results of the most frequently chosen stress coping strategies by engineering students, Table S17. The results of the most frequently chosen stress coping strategies by social sciences students, Table S18. The results of the most frequently chosen stress coping strategies by arts and humanities students, Table S19. The results of the most frequently chosen stress coping strategies by sciences students, Table S20. The results of the most frequently chosen stress coping strategies without the usage of either psychological or psychiatric help before the pandemics outbreak, Table S21. The results of the most frequently chosen stress coping strategies for the usage of psychological help before the pandemics outbreak, Table S22. The results of the most frequently chosen stress coping strategies for the usage of psychological and psychiatric help before the pandemics outbreak, Table S23. The results of the most frequently chosen stress coping strategies for the usage of psychiatric help before the pandemics outbreak, Table S24. The results of the most frequently chosen stress coping strategies for living alone, Table S25. The results of the most frequently chosen stress coping strategies for living with roommates, Table S26. The results of the most frequently chosen stress coping strategies for living with parents, Table S27. The results of the most frequently chosen stress coping strategies for living with partner or spouse, Table S28. The results of the most frequently chosen stress coping strategies for living with partner or spouse and children, Table S29. The results of the most frequently chosen stress coping strategies for change of the lifestyle, Table S30. The results of the most frequently chosen stress coping strategies for changes awaiting the world after the pandemics, Table S31. The results of the most frequently chosen stress coping strategies for fear of infection of the loved ones, Table S32. The results of the most frequently chosen stress coping strategies for financial instability, Table S33. The results of the most frequently chosen stress coping strategies for isolation, Table S34. The results of the most frequently chosen stress coping strategies for loneliness, Table S35. The results of the most frequently chosen stress coping strategies for concern about education, Table S36. The results of the most frequently chosen stress coping strategies for fear of being infected, Table S37. The results of the most frequently chosen stress coping strategies for being not afraid, Table S38. The results of the most frequently chosen stress coping strategies for working mentally, Table S39. The results of the most frequently chosen stress coping strategies for running own business, Table S40. The results of the most frequently chosen stress coping strategies for not working, Table S41. The results of the most frequently chosen stress coping strategies for working physically, Table S42. The results of the most frequently chosen stress coping strategies for having a stable family income and no changes in the economic situation, Table S43. The results of the most frequently chosen stress coping strategies for having barely money for living, Table S44. The results of the most frequently chosen stress coping strategies for having a stable family income, but worse economic situation than before, Table S45. The results of the most frequently chosen stress coping strategies for borrowing money during the outbreak of the pandemic, Table S46. The results of the most frequently chosen stress coping strategies for a need to start using savings, Table S47. The results of using informational support for the field of study, Table S48. The results of venting for the field of study, Table S49. The results of planning for the field of study, Table S50. The results of humor for the field of study, Table S51. The results of acceptance for the field of study, Table S52. The results of religion for the field of study, Table S53. The results of approach coping strategies for difficulties during pandemics, Table S54. The results of avoidant coping strategies for difficulties during pandemics, Table S55. Cumulative number and percentage for DASS depression scale, Table S56. Cumulative number and percentage for DASS anxiety scale, Table S57. Cumulative number and percentage for DASS stress scale, Table S58. The results of active coping on the field of study for women, Table S59. The results of use of informational support on the field of study for women, Table S60. The results of planning on the field of study for women, Table S61. The results of humor on the field of study for women, Table S62. The results of acceptance on the field of study for women, Table S63. The results of religion on the field of study for women, Table S64. The results of self-blame on the field of study for women, Table S65. The results of active coping on the field of study for men.

## Abbreviations

COVID-19	Coronavirus Disease 2019
SARS-CoV-2	Severe Acute Respiratory Syndrome Coronavirus-2
DASS-21	Depression, Anxiety, Stress Scale-21
M	mean
WHO	World Health Organization
