# Supplementary material for: How Do Polish Students Manage Emotional Distress during the COVID-19 Lockdown? A Web-Based Cross-Sectional Study
Source: J Clin Med. 2021 Oct 26;10(21):4964. doi: 10.3390/jcm10214964 (PMC8584627; doi:10.3390/jcm10214964)
Supplement: Supplementary file 1 [file jcm-10-04964-s001.zip › jcm-1383788-supplementary.pdf]

Supplementary Material

Table S1. The results of the DASS total score for the field of study.

	Field of study							
	**Group**	**Mean**	**Median**	**SD**	**Kruskal– Wallis H test**	**Bonferroni correction**	***p*-value**	**Multiple comparisons* (*p*-value)**
**DASS total score**	**I. Arts and humanities**	43.51	39.00	28.98	16.16	0.01	0.0028	II vs. III (0.009)
	**II. Sciences**	46.08	42.00	27.91				
	**III. Medicine**	36.56	31.00	25.49				
	**IV. Engineering**	38.24	32.00	26.79				
	**V. Social sciences**	39.65	35.00	28.14				

*only significant.

Table S2. The results of the DASS depression scale for the field of study.

	Field of study							
	**Group**	**Mean**	**Median**	**SD**	**Kruskal– Wallis H test**	**Bonferroni correction**	***p*-value**	**Multiple comparisons* (*p*-value)**
**DASS Depression Scale**	**I. Arts and humanities**	15.42	13.00	10.92	19.82	0.01	0.0005	II vs. III (0.012)
	**II. Sciences**	16.65	16.00	11.44				
	**III. Medicine**	12.73	10.00	10.01				
	**IV. Engineering**	14.74	12.00	11.15				
	**V. Social sciences**	13.93	12.00	10.80				

*only significant.

Table S3. The results of the DASS anxiety scale for the field of study.

	Field of study							
	**Group**	**Mean**	**Median**	**SD**	**Kruskal– Wallis H test**	**Bonferroni correction**	***p*-value**	**Multiple comparisons***
**DASS Anxiety Scale**	**I. Arts and humanities**	9.02	6.00	9.63	14.00	-	0.0073	-
	**II. Sciences**	9.67	6.00	9.21				
	**III. Medicine**	7.26	4.00	8.00				
	**IV. Engineering**	7.24	4.00	8.07				
	**V. Social sciences**	8.52	6.00	8.64				

*only significant.

Table S4. The results of the DASS stress scale for the field of study.

	Field of study							
	Group	Mean	Median	SD	Kruskal– Wallis H test	Bonferroni correction	*p*-value	Multiple comparisons*
DASS Stress Scale	**I. Arts and humanities**	19.07	19.00	11.63	11.75	-	0.0193	-
	**II. Sciences**	19.77	20.00	11.12				
	**III. Medicine**	16.56	16.00	10.73				
	**IV. Engineering**	16.26	26.00	10.82				
	**V. Social sciences**	17.19	16.00	11.51				

*only significant.

Table S5. The results of the most frequently chosen stress coping strategies for having other chronic disease.

Do you have any chronic disease?				
**Stress coping strategy**	**Other**			
	**N**	**Mean**	**Median**	**SD**
Acceptance	105	2.20	2.50	0.67
Emotional support		1.65	1.50	0.91
Self-distraction		1.54	1.50	0.73
Planning		1.53	1.50	0.78
Venting		1.48	1.50	0.76
Denial		0.44	0.00	0.66
Use of informational support		1.44	1.50	0.84
Positive reframing		1.37	1.50	0.91
Active coping		1.05	1.00	0.75
Humor		1.04	1.00	0.65
Religion		0.86	0.50	0.98
Self-blame		0.82	1.00	0.69
Behavioral disengagement		0.73	0.50	0.72
Substance use		0.33	0.00	0.67

Table S6. The results of the most frequently chosen stress coping strategies without having a chronic disease.

Do you have any chronic disease?				
Stress coping strategy	No			
	N	Mean	Median	SD
Acceptance	1837	2.20	2.50	0.71
Emotional support		1.57	1.50	0.92
Planning		1.42	1.50	0.85
Positive reframing		1.42	1.50	0.90
Self-distraction		1.41	1.50	0.81
Venting		1.23	1.50	0.77
Use of informational support		1.15	1.00	0.86
Humor		1.09	1.00	0.71
Active coping		0.99	1.00	0.80
Religion		0.79	0.50	0.97
Self-blame		0.73	0.50	0.69
Behavioral disengagement		0.69	0.50	0.75
Substance use		0.49	0.00	0.79
Denial		0.42	0.00	0.64

Table S7. The results of the most frequently chosen stress coping strategies for thyroid diseases.

Do you have any chronic disease?				
Stress coping strategy	Thyroid diseases			
	N	Mean	Median	SD
Acceptance	88	2.04	2.00	0.77
Emotional support		1.76	2.00	0.93
Planning		1.51	1.50	0.80
Self-distraction		1.47	1.50	0.75
Positive reframing		1.37	1.25	0.77
Use of informational support		1.36	1.50	0.85
Venting		1.36	1.50	0.70
Active coping		1.00	1.00	0.72
Humor		0.97	1.00	0.61
Self-blame		0.77	0.50	0.76
Behavioral disengagement		0.66	0.50	0.74
Religion		0.60	0.00	0.88
Substance use		0.52	0.00	0.86
Denial		0.39	0.00	0.55

Table S8. The results of the most frequently chosen stress coping strategies for asthma.

Do you have any chronic disease?				
**Stress coping strategy**	**Asthma**			
	**N**	**Mean**	**Median**	**SD**
Acceptance	54	2.23	2.50	0.75
Planning		1.54	1.50	0.82
Positive reframing		1.48	1.50	0.92
Emotional support		1.44	1.50	1.00
Self-distraction		1.36	1.50	0.78
Venting		1.19	1.50	0.74
Use of informational support		1.15	1.00	0.84
Humor		1.08	1.00	0.76
Religion		1.04	1.00	1.07
Active coping		0.93	1.00	0.71
Behavioral disengagement		0.87	0.75	0.77
Self-blame		0.82	1.00	0.73
Substance use		0.62	0.00	0.84
Denial		0.47	0.00	0.75

Table S9. The results of the most frequently chosen stress coping strategies for allergy.

Do you have any chronic disease?				
**Stress coping strategy**	**Allergy**			
	**N**	**Mean**	**Median**	**SD**
Acceptance	32	2.00	2.00	0.80
Emotional support		1.48	1.50	0.96
Self-distraction		1.48	1.50	0.81
Planning		1.39	1.50	0.83
Venting		1.39	1.50	0.77
Positive reframing		1.23	1.50	0.80
Use of informational support		1.22	1.25	0.88
Humor		1.13	1.00	0.61
Active coping		1.09	1.00	0.87
Self-blame		0.98	1.00	0.79
Religion		0.78	0.25	0.97
Behavioral disengagement		0.55	0.00	0.72
Substance use		0.44	0.00	0.74
Denial		0.34	0.20	0.45

Table S10. The results of the most frequently chosen stress coping strategies for diabetes.

Do you have any chronic disease?				
Stress coping strategy	Diabetes			
	N	Mean	Median	SD
Acceptance	15	2.37	2.50	0.64
Emotional support		1.70	2.00	1.03
Venting		1.63	1.50	0.74
Use of informational support		1.60	1.50	0.85
Positive reframing		1.57	2.00	1.10
Planning		1.50	1.50	0.96
Self-distraction		1.43	1.50	0.82
Humor		1.07	1.00	0.62
Active coping		1.03	1.00	0.92
Self-blame		0.90	1.00	0.74
Behavioral disengagement		0.80	1.00	0.84
Religion		0.73	0.00	0.86
Denial		0.53	0.00	0.85
Substance use		0.27	0.00	0.56

Table S11. The results of the most frequently chosen stress coping strategies for mental disorders.

Do you have any chronic disease?				
Stress coping strategy	Mental disorders			
	N	Mean	Median	SD
Acceptance	41	2.22	2.50	0.58
Self-distraction		1.78	2.00	0.76
Venting		1.67	1.50	0.85
Emotional support		1.51	1.50	1.00
Planning		1.48	1.50	0.89
Use of informational support		1.44	1.50	0.88
Humor		1.30	1.50	0.68
Active coping		1.22	1.00	0.96
Positive reframing		1.18	1.00	0.85
Self-blame		1.16	1.00	0.82
Behavioral disengagement		1.12	1.00	0.89
Substance use		1.05	1.00	1.10
Denial		0.67	0.50	0.81
Religion		0.60	0.00	0.87

Table S12. The results of the DASS total score for females and males.

	Females		Males		*p*
	Mean	SD	Mean	SD	
**DASS Total score**	40.54	26.65	31.60	25.02	0.0000001

Table S13. The results of the most frequently chosen type of coping strategies by all respondents.

	N	Mean	Median	Minimum	Maximum	SD	SE
**Avoidant coping**	2172	22.82	22.00	12.00	45.00	5.78	0.12
**Approach coping**	2172	29.60	29.00	12.00	48.00	6.89	0.15

Table S14. The results of the most frequently chosen stress coping strategies by all respondents.

Stress coping strategy	Statement	N	Mean	Median	SD
**Acceptance**	I've been accepting the reality of the fact that it has happened.	2172	2.19	2.50	0.71
	I've been learning to live with it.				
**Emotional support**	I've been getting emotional support from others.		1.58	1.50	0.93
	I've been getting comfort and understanding from someone.				
**Planning**	I've been trying to come up with a strategy about what to do.		1.44	1.50	0.84
	I've been thinking hard about what steps to take.				
**Self-distraction**	I've been turning to work or other activities to take my mind off things.		1.43	1.50	0.80
	I've been doing something to think about it less, such as going to movies, watching TV, reading, daydreaming, sleeping, or shopping.				
**Positive reframing**	I've been trying to see it in a different light, to make it seem more positive.		1.41	1.50	0.90
	I've been looking for something good in what is happening.				
**Venting**	I've been saying things to let my unpleasant feelings escape.		1.28	1.50	0.77
	I've been expressing my negative feelings.				
**Use of informational support**	I’ve been getting help and advice from other people.		1.18	1.00	0.87
	I’ve been trying to get advice or help from other people about what to do.				
**Humor**	I've been making jokes about it.		1.09	1.00	0.71
	I've been making fun of the situation.				
**Active coping**	I've been concentrating my efforts on doing something about the situation I'm in.		1.00	1.00	0.80
	I've been taking action to try to make the situation better.				
**Religion**	I've been trying to find comfort in my religion or spiritual beliefs.		0.79	0.50	0.97
	I've been praying or meditating.				
**Self-blame**	I’ve been criticizing myself.		0.75	0.50	0.70
	I’ve been blaming myself for things that happened.				
**Behavioral disengagement**	I've been giving up trying to deal with it.		0.70	0.50	0.76
	I've been giving up the attempt to cope.				
**Substance use**	I've been using alcohol or other drugs to make myself feel better.		0.50	0.00	0.80
	I've been using alcohol or other drugs to help me get through it.				
**Denial**	I've been saying to myself "this isn't real".		0.43	0.00	0.64
	I've been refusing to believe that it has happened.				

Table S15. The results of the most frequently chosen stress coping strategies by medical students.

Stress coping strategy	Medicine		
	**Mean**	**Median**	**SD**
Acceptance	2.26	2.50	0.68
Emotional support	1.62	1.50	0.91
Planning	1.50	1.50	0.83
Positive reframing	1.45	1.50	0.89
Self-distraction	1.44	1.50	0.80
Venting	1.30	1.50	0.74
Use of informational support	1.24	1.00	0.85
Humor	1.13	1.00	0.73
Active coping	1.05	1.00	0.81
Religion	0.86	0.50	0.99
Self-blame	0.77	0.50	0.69
Behavioral disengagement	0.67	0.50	0.73
Substance use	0.46	0.00	0.74
Denial	0.40	0.00	0.62

Table S16. The results of the most frequently chosen stress coping strategies by engineering students.

Stress coping strategy	Engineering		
	**Mean**	**Median**	**SD**
Acceptance	2.18	2.50	0.73
Planning	1.39	1.50	0.87
Emotional support	1.39	1.50	0.94
Self-distraction	1.33	1.50	0.78
Positive reframing	1.28	1.00	0.90
Humor	1.13	1.00	0.71
Venting	1.10	1.00	0.78
Use of informational support	0.99	1.00	0.77
Active coping	0.93	1.00	0.77
Self-blame	0.75	0.50	0.75
Behavioral disengagement	0.67	0.50	0.79
Religion	0.63	0.00	0.90
Substance use	0.56	0.00	0.89
Denial	0.41	0.00	0.65

Table S17. The results of the most frequently chosen stress coping strategies by social sciences students.

Stress coping strategy	Social sciences		
	**Mean**	**Median**	**SD**
Acceptance	2.04	2.00	0.74
Emotional support	1.54	1.50	0.90
Self-distraction	1.43	1.50	0.82
Positive reframing	1.39	1.50	0.89
Planning	1.31	1.00	0.85
Venting	1.25	1.50	0.83
Use of informational support	1.13	1.00	0.88
Active coping	0.95	1.00	0.73
Humor	0.94	1.00	0.65
Behavioral disengagement	0.76	0.50	0.80
Self-blame	0.67	0.50	0.69
Religion	0.66	0.00	0.91
Substance use	0.57	0.00	0.89
Denial	0.52	0.00	0.71

Table S18. The results of the most frequently chosen stress coping strategies by arts and humanities students.

Stress coping strategy	Arts and humanities		
	Mean	Median	SD
Acceptance	2.10	2.00	0.76
Emotional support	1.49	1.50	1.00
Self-distraction	1.35	1.50	0.85
Planning	1.33	1.50	0.82
Positive reframing	1.31	1.25	0.94
Venting	1.26	1.50	0.86
Humor	1.09	1.00	0.68
Use of informational support	1.05	1.00	0.98
Active coping	0.92	1.00	0.82
Behavioral disengagement	0.79	0.50	0.82
Religion	0.74	0.00	0.99
Self-blame	0.70	0.50	0.71
Substance use	0.57	0.00	0.87
Denial	0.45	0.00	0.65

Table S19. The results of the most frequently chosen stress coping strategies by sciences students.

Stress coping strategy	Sciences		
	Mean	Median	SD
Acceptance	2.02	2.00	0.82
Emotional support	1.53	1.50	0.98
Self-distraction	1.42	1.50	0.82
Venting	1.41	1.50	0.78
Positive reframing	1.33	1.00	0.90
Planning	1.26	1.00	0.91
Use of informational support	1.17	1.00	0.91
Humor	1.07	1.00	0.63
Active coping	0.88	1.00	0.78
Self-blame	0.83	0.50	0.69
Behavioral disengagement	0.82	0.50	0.79
Religion	0.69	0.00	0.93
Substance use	0.56	0.00	0.80
Denial	0.47	0.00	0.61

Table S20. The results of the most frequently chosen stress coping strategies without the usage of either psychological or psychiatric help before the pandemics outbreak.

Did you use psychological/psychiatric help before the beginning of the pandemics?				
Stress coping strategy	No			
	N	Mean	Median	SD
Acceptance	1800	2.19	2.50	0.71
Emotional support		1.57	1.50	0.92
Planning		1.42	1.50	0.84
Positive reframing		1.40	1.50	0.89
Self-distraction		1.40	1.50	0.81
Venting		1.24	1.50	0.76
Use of informational support		1.14	1.00	0.85
Humor		1.07	1.00	0.70
Active coping		0.98	1.00	0.78
Religion		0.80	0.50	0.98
Self-blame		0.70	0.50	0.67
Behavioral disengagement		0.66	0.50	0.73
Substance use		0.46	0.00	0.77
Denial		0.43	0.00	0.64

Table S21. The results of the most frequently chosen stress coping strategies for the usage of psychological help before the pandemics outbreak.

Did you use psychological/psychiatric help before the beginning of the pandemics?				
Stress coping strategy	Yes, I used psychological services			
	N	Mean	Median	SD
Acceptance	162	2.25	2.50	0.71
Emotional support		1.73	2.00	0.90
Planning		1.70	1.50	0.82
Self-distraction		1.66	1.50	0.71
Positive reframing		1.64	1.50	0.88
Venting		1.58	1.50	0.74
Use of informational support		1.49	1.50	0.89
Active coping		1.34	1.25	0.82
Humor		1.15	1.00	0.70
Self-blame		0.96	1.00	0.71
Religion		0.89	0.50	1.01
Behavioral disengagement		0.81	0.75	0.75
Substance use		0.48	0.00	0.76
Denial		0.42	0.00	0.63

Table S22. The results of the most frequently chosen stress coping strategies for the usage of psychological and psychiatric help before the pandemics outbreak.

Did you use psychological/psychiatric help before the beginning of the pandemics?				
**Stress coping strategy**	**Yes, I used psychological and psychiatric services**			
	**N**	**Mean**	**Median**	**SD**
Acceptance	130	2.17	2.00	0.71
Emotional support		1.52	1.50	0.99
Self-distraction		1.52	1.50	0.83
Venting		1.42	1.50	0.82
Use of informational support		1.33	1.50	0.93
Positive reframing		1.31	1.00	0.96
Planning		1.28	1.00	0.89
Humor		1.15	1.00	0.71
Self-blame		1.09	1.00	0.87
Behavioral disengagement		1.02	1.00	0.89
Active coping		0.90	1.00	0.81
Substance use		0.78	0.00	0.99
Religion		0.53	0.00	0.82
Denial		0.39	0.00	0.66

Table S23. The results of the most frequently chosen stress coping strategies for the usage of psychiatric help before the pandemics outbreak.

Did you use psychological/psychiatric help before the beginning of the pandemics?				
**Stress coping strategy**	**Yes, I used psychiatric services**			
	**N**	**Mean**	**Median**	**SD**
Acceptance	80	2.19	2.50	0.76
Emotional support		1.45	1.50	0.91
Planning		1.44	1.50	0.88
Self-distraction		1.41	1.50	0.73
Humor		1.31	1.50	0.74
Venting		1.28	1.25	0.79
Use of informational support		1.24	1.00	0.88
Positive reframing		1.23	1.00	0.93
Self-blame		1.01	1.00	0.89
Active coping		0.96	1.00	0.88
Behavioral disengagement		0.94	0.75	0.89
Substance use		0.87	0.50	1.00
Religion		0.60	0.00	0.83
Denial		0.43	0.00	0.65

Table S24. The results of the most frequently chosen stress coping strategies for living alone.

I live with:				
Stress coping strategy	Alone			
	N	Mean	Median	SD
Acceptance	231	2.26	2.50	0.77
Planning		1.60	1.50	0.89
Positive reframing		1.58	1.50	0.99
Emotional support		1.58	1.50	0.99
Self-distraction		1.47	1.50	0.85
Venting		1.35	1.50	0.82
Humor		1.27	1.00	0.77
Use of informational support		1.21	1.00	0.94
Active coping		1.17	1.00	0.93
Religion		0.89	0.50	1.02
Self-blame		0.83	1.00	0.71
Behavioral disengagement		0.78	0.50	0.82
Substance use		0.71	0.50	0.83
Denial		0.51	0.00	0.66

Table S25. The results of the most frequently chosen stress coping strategies for living with roommates.

I live with:				
Stress coping strategy	Roommates			
	N	Mean	Median	SD
Acceptance	565	2.21	2.50	0.67
Emotional support		1.60	1.50	0.92
Planning		1.47	1.50	0.85
Self-distraction		1.45	1.50	0.80
Positive reframing		1.44	1.50	0.89
Venting		1.33	1.50	0.77
Use of informational support		1.23	1.00	0.86
Humor		1.10	1.00	0.70
Active coping		0.98	1.00	0.78
Religion		0.85	0.50	0.97
Self-blame		0.82	0.50	0.70
Behavioral disengagement		0.74	0.50	0.77
Substance use		0.61	0.00	0.88
Denial		0.46	0.00	0.67

Table S26. The results of the most frequently chosen stress coping strategies for living with parents.

Stress coping strategy	I live with: Parents			
	N	Mean	Median	SD
Acceptance	1049	2.16	2.00	0.72
Emotional support		1.49	1.50	0.92
Self-distraction		1.43	1.50	0.78
Planning		1.38	1.50	0.83
Positive reframing		1.37	1.50	0.89
Venting		1.26	1.50	0.76
Use of informational support		1.14	1.00	0.86
Humor		1.08	1.00	0.70
Active coping		0.96	1.00	0.78
Religion		0.82	0.50	1.00
Self-blame		0.75	0.50	0.71
Behavioral disengagement		0.71	0.50	0.76
Substance use		0.41	0.00	0.73
Denial		0.40	0.00	0.63

Table S27. The results of the most frequently chosen stress coping strategies for living with partner or spouse.

Stress coping strategy	I live with: Partner or spouse			
	N	Mean	Median	SD
Acceptance	301	2.24	2.50	0.70
Emotional support		1.81	2.00	0.90
Planning		1.44	1.50	0.84
Positive reframing		1.39	1.50	0.86
Self-distraction		1.32	1.50	0.85
Use of informational support		1.25	1.00	0.85
Venting		1.19	1.00	0.80
Active coping		1.06	1.00	0.78
Humor		1.00	1.00	0.67
Self-blame		0.60	0.50	0.65
Behavioral disengagement		0.53	0.50	0.65
Religion		0.51	0.00	0.78
Substance use		0.44	0.00	0.78
Denial		0.36	0.00	0.59

Table S28. The results of the most frequently chosen stress coping strategies for living with partner or spouse and children.

I live with:				
**Stress coping strategy**	**Partner or spouse and children**			
	**N**	**Mean**	**Median**	**SD**
Acceptance	21	2.12	2.50	0.71
Emotional support		1.86	1.50	0.81
Self-distraction		1.38	1.50	0.76
Positive reframing		1.38	1.50	0.71
Planning		1.57	2.00	0.86
Venting		1.19	1.00	0.56
Use of informational support		1.05	1.00	0.63
Active coping		0.90	1.00	0.64
Humor		0.67	0.50	0.64
Religion		0.64	0.00	0.95
Behavioral disengagement		0.48	0.00	0.64
Denial		0.40	0.00	0.54
Self-blame		0.36	0.00	0.50
Substance use		0.19	0.00	0.51

Table S29. The results of the most frequently chosen stress coping strategies for change of the lifestyle.

What was the most difficult for you during pandemics?				
**Coping stress strategy**	**Change of the lifestyle**			
	**N**	**Mean**	**Median**	**SD**
Acceptance	312	2.15	2.50	0.74
Emotional support		1.46	1.50	0.95
Self-distraction		1.36	1.50	0.80
Planning		1.35	1.50	0.86
Positive reframing		1.31	1.50	0.84
Venting		1.29	1.50	0.81
Humor		1.13	1.00	0.69
Use of informational support		1.12	1.00	0.90
Active coping		0.87	1.00	0.74
Behavioral disengagement		0.80	0.50	0.84
Religion		0.72	0.00	0.97
Self-blame		0.71	0.50	0.69
Substance use		0.53	0.00	0.82
Denial		0.41	0.00	0.65

Table S30. The results of the most frequently chosen stress coping strategies for changes awaiting the world after the pandemics.

What was the most difficult for you during pandemics?				
Coping stress strategy	Changes awaiting the world after the pandemics			
	N	Mean	Median	SD
Acceptance	446	2.28	2.50	0.68
Emotional support		1.66	1.50	0.92
Planning		1.59	1.50	0.87
Positive reframing		1.44	1.50	0.93
Self-distraction		1.44	1.50	0.81
Venting		1.26	1.50	0.76
Use of informational support		1.21	1.00	0.88
Humor		1.14	1.00	0.69
Active coping		1.02	1.00	0.81
Religion		0.75	0.00	0.96
Self-blame		0.73	0.50	0.69
Behavioral disengagement		0.66	0.50	0.74
Substance use		0.47	0.00	0.77
Denial		0.33	0.00	0.56

Table S31. The results of the most frequently chosen stress coping strategies for fear of infection of the loved ones.

What was the most difficult for you during pandemics?				
Coping stress strategy	Fear of infection of the loved ones			
	N	Mean	Median	SD
Acceptance	730	2.21	2.50	0.67
Emotional support		1.70	2.00	0.90
Self-distraction		1.49	1.50	0.80
Planning		1.45	1.50	0.79
Positive reframing		1.45	1.50	0.85
Venting		1.29	1.50	0.75
Use of informational support		1.26	1.00	0.85
Active coping		1.05	1.00	0.78
Humor		0.95	1.00	0.66
Religion		0.93	1.00	0.99
Self-blame		0.72	0.50	0.66
Behavioral disengagement		0.63	0.50	0.69
Denial		0.45	0.00	0.63
Substance use		0.43	0.00	0.72

Table S32. The results of the most frequently chosen stress coping strategies for financial instability.

What was the most difficult for you during pandemics?				
Coping stress strategy	Financial instability			
	N	Mean	Median	SD
Acceptance	157	2.17	2.00	0.75
Emotional support		1.57	1.50	0.97
Planning		1.42	1.50	0.91
Self-distraction		1.37	1.50	0.85
Positive reframing		1.32	1.00	0.97
Humor		1.20	1.00	0.77
Venting		1.20	1.50	0.80
Use of informational support		1.08	1.00	0.85
Active coping		1.02	1.00	0.86
Self-blame		0.81	0.50	0.80
Behavioral disengagement		0.79	0.50	0.81
Religion		0.61	0.00	0.93
Substance use		0.56	0.00	0.86
Denial		0.46	0.00	0.65

Table S33. The results of the most frequently chosen stress coping strategies for isolation.

What was the most difficult for you during pandemics?				
Coping stress strategy	Isolation			
	N	Mean	Median	SD
Acceptance	224	2.15	2.50	0.74
Emotional support		1.50	1.50	0.93
Positive reframing		1.46	1.50	0.89
Planning		1.42	1.50	0.82
Self-distraction		1.41	1.50	0.75
Venting		1.29	1.50	0.74
Use of informational support		1.15	1.00	0.84
Humor		1.16	1.00	0.71
Active coping		1.01	1.00	0.80
Self-blame		0.71	0.50	0.65
Religion		0.67	0.00	0.91
Substance use		0.61	0.00	0.89
Behavioral disengagement		0.61	0.50	0.71
Denial		0.44	0.00	0.68

Table S34. The results of the most frequently chosen stress coping strategies for loneliness.

What was the most difficult for you during pandemics?				
Coping stress strategy	Loneliness			
	N	Mean	Median	SD
Acceptance	158	2.06	2.00	0.75
Self-distraction		1.49	1.50	0.71
Venting		1.46	1.50	0.73
Positive reframing		1.30	1.00	0.91
Emotional support		1.29	1.00	0.83
Planning		1.27	1.00	0.81
Use of informational support		1.14	1.00	0.83
Self-blame		1.13	1.00	0.77
Humor		1.12	1.00	0.72
Behavioral disengagement		1.00	1.00	0.84
Active coping		0.99	1.00	0.78
Religion		0.68	0.00	0.90
Substance use		0.67	0.00	0.89
Denial		0.55	0.00	0.73

Table S35. The results of the most frequently chosen stress coping strategies for concern about education.

What was the most difficult for you during pandemics?				
Coping stress strategy	Concern about education			
	N	Mean	Median	SD
Acceptance	47	2.20	2.50	0.83
Positive reframing		1.48	1.50	0.99
Planning		1.37	1.50	0.93
Emotional support		1.30	1.50	0.96
Humor		1.23	1.50	0.77
Self-distraction		1.04	1.00	0.80
Use of informational support		1.00	1.00	0.90
Venting		0.94	1.00	0.75
Active coping		0.91	1.00	0.82
Self-blame		0.73	0.50	0.74
Religion		0.64	0.00	0.91
Behavioral disengagement		0.51	0.50	0.62
Substance use		0.31	0.00	0.61
Denial		0.16	0.00	0.31

Table S36. The results of the most frequently chosen stress coping strategies for fear of being infected.

What was the most difficult for you during pandemics?				
**Coping stress strategy**	**Fear of being infected**			
	**N**	**Mean**	**Median**	**SD**
Acceptance	69	2.06	2.00	0.70
Emotional support		1.63	1.50	0.80
Self-distraction		1.59	2.00	0.86
Positive reframing		1.50	1.50	1.01
Planning		1.43	1.50	0.85
Venting		1.41	1.50	0.86
Use of informational support		1.22	1.00	0.81
Active coping		1.12	1.00	0.81
Religion		1.06	1.00	1.09
Humor		1.04	1.00	0.73
Behavioral disengagement		0.88	1.00	0.86
Denial		0.80	0.50	0.80
Self-blame		0.74	0.50	0.72
Substance use		0.56	0.00	0.93

Table S37. The results of the most frequently chosen stress coping strategies for being not afraid.

What was the most difficult for you during pandemics?				
**Coping stress strategy**	**I was not afraid**			
	**N**	**Mean**	**Median**	**SD**
Acceptance	26	2.40	2.50	0.84
Humor		1.85	1.75	0.96
Positive reframing		1.21	1.25	1.12
Emotional support		0.85	0.00	1.16
Planning		0.73	0.50	0.96
Use of informational support		0.56	0.00	0.92
Venting		0.50	0.00	0.77
Self-distraction		0.48	0.00	0.75
Religion		0.46	0.00	0.89
Active coping		0.38	0.00	0.79
Substance use		0.31	0.00	0.72
Behavioral disengagement		0.23	0.00	0.32
Self-blame		0.19	0.00	0.38
Denial		0.06	0.00	0.22

Table S38. The results of the most frequently chosen stress coping strategies for working mentally.

Stress coping strategy	Are you currently working?			
	I work mentally			
	N	Mean	Median	SD
Acceptance	249	2.20	2.50	0.73
Emotional support		1.55	1.50	0.93
Positive reframing		1.51	1.50	0.90
Self-distraction		1.48	1.50	0.88
Planning		1.46	1.50	0.90
Active coping		1.15	1.00	0.85
Venting		1.16	1.00	0.80
Use of informational support		1.10	1.00	0.86
Humor		1.07	1.00	0.68
Religion		0.78	0.00	0.97
Self-blame		0.58	0.50	0.63
Behavioral disengagement		0.53	0.00	0.70
Substance use		0.51	0.00	0.81
Denial		0.42	0.00	0.65

Table S39. The results of the most frequently chosen stress coping strategies for running own business.

Stress coping strategy	Are you currently working?			
	I run my own business			
	N	Mean	Median	SD
Acceptance	28	2.34	2.50	0.77
Planning		1.80	2.00	0.90
Positive reframing		1.70	1.50	0.92
Emotional support		1.52	1.50	1.00
Self-distraction		1.50	1.50	0.77
Humor		1.34	1.25	0.78
Active coping		1.23	1.00	0.98
Venting		1.25	1.00	0.88
Use of informational support		1.20	1.00	1.02
Religion		0.70	0.00	1.07
Substance use		0.66	0.00	1.06
Denial		0.46	0.00	0.82
Self-blame		0.45	0.00	0.60
Behavioral disengagement		0.38	0.00	0.68

Table S40. The results of the most frequently chosen stress coping strategies for not working.

Stress coping strategy	Are you currently working?			
	No, I do not work			
	N	Mean	Median	SD
Acceptance	1799	2.19	2.50	0.70
Emotional support		1.58	1.50	0.93
Planning		1.44	1.50	0.84
Self-distraction		1.42	1.50	0.79
Positive reframing		1.40	1.50	0.90
Venting		1.30	1.50	0.77
Use of informational support		1.20	1.00	0.86
Humor		1.09	1.00	0.71
Active coping		0.97	1.00	0.78
Religion		0.79	0.50	0.97
Self-blame		0.79	0.50	0.71
Behavioral disengagement		0.73	0.50	0.76
Substance use		0.48	0.00	0.78
Denial		0.42	0.00	0.63

Table S41. The results of the most frequently chosen stress coping strategies for working physically.

Stress coping strategy	Are you currently working?			
	I work physically			
	N	Mean	Median	SD
Acceptance	96	2.16	2.00	0.76
Emotional support		1.59	1.50	0.92
Self-distraction		1.41	1.50	0.78
Planning		1.28	1.25	0.77
Positive reframing		1.28	1.00	0.87
Venting		1.13	1.00	0.68
Use of informational support		1.12	1.00	0.85
Active coping		1.09	1.00	0.82
Humor		1.07	1.00	0.69
Substance use		0.78	1.00	0.88
Religion		0.70	0.25	0.88
Self-blame		0.67	0.50	0.69
Behavioral disengagement		0.63	0.50	0.70
Denial		0.50	0.00	0.70

Table S42. The results of the most frequently chosen stress coping strategies for having a stable family income and no changes in the economic situation.

Stress coping strategy	How do you assess your economic situation during pandemics?			
	I have a stable family income, nothing has changed			
	N	Mean	Median	SD
Acceptance	1278	2.22	2.50	0.70
Emotional support		1.55	1.50	0.94
Positive reframing		1.43	1.50	0.90
Planning		1.37	1.50	0.85
Self-distraction		1.36	1.50	0.81
Venting		1.21	1.00	0.76
Use of informational support		1.15	1.00	0.87
Humor		1.11	1.00	0.72
Active coping		0.93	1.00	0.78
Religion		0.81	0.50	0.97
Self-blame		0.69	0.50	0.68
Behavioral disengagement		0.62	0.50	0.72
Substance use		0.44	0.00	0.74
Denial		0.37	0.00	0.60

Table S43. The results of the most frequently chosen stress coping strategies for having barely money for living.

Stress coping strategy	How do you assess your economic situation during pandemics?			
	I barely have enough money for living			
	N	Mean	Median	SD
Acceptance	31	1.81	2.00	0.93
Self-distraction		1.47	1.50	0.87
Emotional support		1.39	1.00	1.03
Positive reframing		1.34	1.50	0.93
Venting		1.31	1.00	0.87
Self-blame		1.27	1.00	1.06
Planning		1.24	1.00	0.87
Behavioral disengagement		1.19	1.00	1.02
Use of informational support		1.02	1.00	0.79
Active coping		0.95	1.00	0.87
Humor		0.95	1.00	0.75
Substance use		0.84	0.00	1.07
Denial		0.82	1.00	0.83
Religion		0.69	0.00	1.03

Table S44. The results of the most frequently chosen stress coping strategies for having a stable family income, but worse economic situation than before

	How do you assess your economic situation during pandemics?			
**Stress coping strategy**	**I have a stable family income, but the situation is worse than before**			
	**N**	**Mean**	**Median**	**SD**
Acceptance	647	2.19	2.00	0.69
Emotional support		1.63	1.50	0.88
Self-distraction		1.53	1.50	0.78
Planning		1.52	1.50	0.81
Positive reframing		1.41	1.50	0.86
Venting		1.37	1.50	0.77
Use of informational support		1.25	1.00	0.85
Active coping		1.09	1.00	0.79
Humor		1.06	1.00	0.66
Self-blame		0.80	0.50	0.71
Religion		0.77	0.50	0.96
Behavioral disengagement		0.76	0.50	0.76
Substance use		0.48	0.00	0.78
Denial		0.46	0.00	0.65

Table S45. The results of the most frequently chosen stress coping strategies for borrowing money during the outbreak of the pandemic

How do you assess your economic situation during pandemics?				
Stress coping strategy	I have to borrow money from my family/friends during the outbreak of the pandemic			
	N	Mean	Median	SD
Acceptance	25	1.92	2.00	0.83
Planning		1.52	1.50	1.02
Self-distraction		1.48	1.50	0.78
Behavioral disengagement		1.38	1.50	0.71
Venting		1.28	1.00	0.93
Active coping		1.14	1.00	0.94
Emotional support		1.14	1.00	1.07
Positive reframing		1.12	1.00	0.98
Substance use		1.10	1.00	0.92
Humor		1.10	1.00	0.87
Self-blame		1.06	1.00	0.74
Use of informational support		0.94	0.50	0.95
Religion		0.78	0.00	1.08
Denial		0.76	0.50	0.71

Table S46. The results of the most frequently chosen stress coping strategies for a need to start using savings.

How do you assess your economic situation during pandemics?				
Stress coping strategy	I have to start using savings			
	N	Mean	Median	SD
Acceptance	191	2.11	2.00	0.78
Emotional support		1.67	2.00	0.93
Planning		1.61	1.50	0.88
Self-distraction		1.51	1.50	0.79
Venting		1.38	1.50	0.79
Positive reframing		1.35	1.50	0.95
Use of informational support		1.26	1.00	0.86
Active coping		1.14	1.00	0.82
Humor		1.08	1.00	0.73
Behavioral disengagement		0.85	1.00	0.82
Self-blame		0.85	1.00	0.72
Substance use		0.79	0.00	1.02
Religion		0.72	0.00	0.96
Denial		0.59	0.00	0.76

Table S47. The results of using informational support for the field of study.

Use of informational support								
**Field of study**	**Mean**	**Median**	**SD**	**Kruskal– Wallis H test**	**Bonferroni correction**	***p*-value**	**z-value**	**Multiple comparisons* (*p*-value)**
**I. Arts and humanities**	1.05	1.00	0.98	20.74	0.01	0.0004	3.70	III vs. IV (0.002)
**II. Sciences**	1.17	1.00	0.91					
**III. Medicine**	1.24	1.00	0.85					
**IV. Engineering**	0.99	1.00	0.77					
**V. Social sciences**	1.13	1.00	0.88					

*only significant.

Table S48. The results of venting for the field of study.

Venting								
**Field of study**	**Mean**	**Median**	**SD**	**Kruskal– Wallis H test**	**Bonferroni correction**	***p*-value**	**z-value**	**Multiple comparisons* (*p*-value)**
**I. Arts and humanities**	1.26	1.50	0.86	18.39	0.01	0.0010	II vs. IV 3.35 III vs. IV 3.83	II vs. IV (0.008) III vs. IV (0.001)
**II. Sciences**	1.41	1.50	0.78					
**III. Medicine**	1.30	1.50	0.74					
**IV. Engineering**	1.10	1.00	0.78					
**V. Social sciences**	1.25	1.50	0.83					

*only significant.

Table S49. The results of planning for the field of study.

Planning								
**Field of study**	**Mean**	**Median**	**SD**	**Kruskal– Wallis H test**	**Bonferroni correction**	***p*-value**	**z-value**	**Multiple comparisons* (*p*-value)**
**I. Arts and humanities**	1.33	1.50	0.82	25.30	0.01	0.00001	4.16	III vs. V (0.0001)
**II. Sciences**	1.26	1.00	0.91					
**III. Medicine**	1.50	1.50	0.83					
**IV. Engineering**	1.39	1.50	0.87					
**V. Social sciences**	1.31	1.00	0.85					

*only significant.

Table S50. The results of humor for the field of study.

Humor								
Field of study	Mean	Median	SD	Kruskal– Wallis H test	Bonferroni correction	*p*-value	z-value	Multiple comparisons* (*p*-value)
**I. Arts and humanities**	1.09	1.00	0.68	22.39	0.01	0.0002	III vs. V 4.38 IV vs. V 3.40	III vs. V (0.0001) IV vs. V (0.007)
**II. Sciences**	1.07	1.00	0.63					
**III. Medicine**	1.13	1.00	0.73					
**IV. Engineering**	1.13	1.00	0.71					
**V. Social sciences**	0.94	1.00	0.65					

*only significant.

Table S51. The results of acceptance for the field of study.

Acceptance								
Field of study	Mean	Median	SD	Kruskal– Wallis H test	Bonferroni correction	*p*-value	z-value	Multiple comparisons* (*p*-value)
**I. Arts and humanities**	2.10	2.00	0.76	37.08	0.01	0.00001	5.45	III vs. V (0.0001)
**II. Sciences**	2.02	2.00	0.82					
**III. Medicine**	2.26	2.50	0.68					
**IV. Engineering**	2.18	2.50	0.73					
**V. Social sciences**	2.04	2.00	0.74					

*only significant.

Table S52. The results of religion for the field of study.

Religion								
Field of study	Mean	Median	SD	Kruskal– Wallis H test	Bonferroni correction	*p*-value	z-value	Multiple comparisons* (*p*-value)
**I. Arts and humanities**	0.74	0.00	0.99	27.07	0.01	0.00001	3.95	III vs. V (0.001)
**II. Sciences**	0.69	0.00	0.93					
**III. Medicine**	0.86	0.50	0.99					
**IV. Engineering**	0.63	0.00	0.90					
**V. Social sciences**	0.66	0.00	0.91					

*only significant.

Table S53. The results of approach coping strategies for difficulties during pandemics.

Approach coping				
**What was the most difficult for you during pandemics?**				
	**Kruskal– Wallis H test**	***p*-value**	**z-value**	**Multiple comparisons* (*p*-value)**
**I. Isolation**	43.75	0.00001	VIII vs. II 3.92<br>VIII vs. IX 3.88<br>IV vs. IX 3.84	VIII vs. II (0.003)<br>VIII vs. IX (0.004)<br>IV vs. IX (0.004)
**II. Fear of infection of the loved ones**				
**III. Concern about education**				
**IV. I was not afraid**				
**V. Financial unstability**				
**VI. Fear of being infected**				
**VII. Change of the lifestyle**				
**VIII. Loneliness**				
**IX. Changes awaiting the world after the pandemics**				

*only significant.

Table S54. The results of avoidant coping strategies for difficulties during pandemics.

Avoidant coping				
**What was the most difficult for you during pandemics?**				
	**Kruskal– Wallis H test**	***p*-value**	**z-value**	**Multiple comparisons* (*p*-value)**
**I. Isolation**	79.18	0.00001	VIII vs. IV 7.29<br>VI vs. IV 5.91<br>III vs. VIII 5.48	VIII vs. IV (0.0001)<br>VI vs. IV (0.0001)<br>III vs. VIII (0.0001)
**II. Fear of infection of the loved ones**				
**III. Concern about education**				
**IV. I was not afraid**				
**V. Financial unstability**				
**VI. Fear of being infected**				
**VII. Change of the lifestyle**				
**VIII. Loneliness**				
**IX. Changes awaiting the world after the pandemics**				

*only significant.

Table S55. Cumulative number and percentage for DASS depression scale.

		DASS depression scale			
	Class	Number	Cumulative number	Percent	Cumulative percentage
**Normal**	0	127	127	5.85	5.85
	1	171	298	7.87	13.72
	2	236	534	10.87	24.59
	3	231	765	10.64	35.22
	4	183	948	8.43	43.65
**Mild**	5	150	1098	6.91	50.55
	6	132	1230	6.08	56.63
**Moderate**	7	120	1350	5.52	62.15
	8	126	1476	5.80	67.96
	9	108	1584	4.97	72.93
	10	78	1662	3.59	76.52
**Severe**	11	72	1734	3.31	79.83
	12	73	1807	3.36	83.20
	13	76	1883	3.50	86.69
**Extremely severe**	14	65	1948	2.99	89.69
	15	59	2007	2.72	92.40
	16	43	2050	1.98	94.38
	17	24	2074	1.10	95.49
	18	30	2104	1.38	96.87
	19	21	2125	0.97	97.84
	20	29	2154	1.34	99.17
	21	18	2172	0.83	100.00
		0	2172	0.00	100.00

Table S56. Cumulative number and percentage for DASS anxiety scale.

	Class	DASS anxiety scale			
		Number	Cumulative number	Percent	Cumulative percentage
**Normal**	0	527	527	24.26	24.26
	1	317	844	14.59	38.86
	2	250	1094	11.51	50.37
	3	213	1307	9.81	60.18
**Mild**	4	133	1440	6.12	66.30
	5	140	1580	6.45	72.74
**Moderate**	6	108	1688	4.97	77.72
	7	87	1775	4.01	81.72
**Severe**	8	81	1856	3.73	85.45
	9	63	1919	2.90	88.35
**Extremely severe**	10	57	1976	2.62	90.98
	11	50	2026	2.30	93.28
	12	38	2064	1.75	95.03
	13	26	2090	1.20	96.22
	14	26	2116	1.20	97.42
	15	15	2131	0.69	98.11
	16	13	2144	0.60	98.71
	17	16	2160	0.74	99.45
	18	6	2166	0.28	99.72
	19	3	2169	0.14	99.86
	20	3	2172	0.14	100.00
		0	2172	0.00	100.00

Table S57. Cumulative number and percentage for DASS stress scale.

	Class	DASS stress scale			
		Number	Cumulative number	Percent	Cumulative percentage
**Normal**	0	141	141	6.49	6.49
	1	102	243	4.70	11.19
	2	116	359	5.34	16.53
	3	121	480	5.57	22.10
	4	132	612	6.08	28.18
	5	125	737	5.76	33.93
	6	147	884	6.77	40.70
	7	142	1026	6.54	47.24
**Mild**	8	126	1152	5.80	53.04
	9	129	1281	5.94	58.98
**Moderate**	10	119	1400	5.48	64.46
	11	106	1506	4.88	69.34
	12	108	1614	4.97	74.31
**Severe**	13	107	1721	4.93	79.24
	14	90	1811	4.14	83.38
	15	96	1907	4.42	87.80
	16	71	1978	3.27	91.07
**Extremely severe**	17	60	2038	2.76	93.83
	18	40	2078	1.84	95.67
	19	39	2117	1.80	97.47
	20	26	2143	1.20	98.66
	21	29	2172	1.34	100.00
		0	2172	0.00	100.00

Table S58. The results of active coping on the field of study for women.

Women										
**Active coping**										
**Field of study**	**Mean**	**Median**	**SD**	**SE**	**R**	**Kruskal– Wallis H test**	**Bonferroni correction**	***p*-value**	**z-value***	**Multiple comparisons* (*p*-value)**
**I. Arts and humanities**	0.92	1.00	0.80	0.09	727.62					
**II. Sciences**	0.89	1.00	0.77	0.08	715.28					
**III. Medicine**	1.07	1.00	0.81	0.03	813.05	12.64	0.01	0.0132	III vs. V 2.81	III vs. V 0.05
**IV. Engineering**	1.08	1.00	0.79	0.08	823.67					
**V. Social sciences**	0.91	1.00	0.72	0.04	732.45					

*only significant.

Table S59. The results of use of informational support on the field of study for women.

Women										
**Use of informational support**										
**Field of study**	**Mean**	**Median**	**SD**	**SE**	**R**	**Kruskal– Wallis H test**	**Bonferroni correction**	***p*-value**	**z-value***	**Multiple comparisons* (*p*-value)**
**I. Arts and humanities**	1.10	1.00	1.00	0.11	687.90					
**II. Sciences**	1.20	1.00	0.91	0.10	750.27					
**III. Medicine**	1.33	1.50	0.85	0.03	819.84	14.86	0.01	0.0050	III vs. V 2.96	III vs. V 0.03
**IV. Engineering**	1.19	1.00	0.81	0.08	750.60					
**V. Social sciences**	1.17	1.00	0.88	0.05	734.97					

*only significant.

Table S60. The results of planning on the field of study for women.

Women										
**Planning**										
**Field of study**	**Mean**	**Median**	**SD**	**SE**	**R**	**Kruskal– Wallis H test**	**Bonferroni correction**	***p*-value**	**z-value***	**Multiple comparisons* (*p*-value)**
**I. Arts and humanities**	1.39	1.50	0.78	0.09	750.49					
**II. Sciences**	1.27	1.50	0.92	0.10	693.46					
**III. Medicine**	1.53	1.50	0.82	0.03	828.58	25.77	0.01	0.00001	III vs. V 4.53	III vs. V 0.00006
**IV. Engineering**	1.45	1.50	0.82	0.08	786.38					
**V. Social sciences**	1.29	1.00	0.85	0.05	698.47					

*only significant.

Table S61. The results of humor on the field of study for women.

Women										
Humor										
**Field of study**	**Mean**	**Median**	**SD**	**SE**	**R**	**Kruskal– Wallis H test**	**Bonferroni correction**	***p*-value**	**z-value***	**Multiple comparisons* (*p*-value)**
**I. Arts and humanities**	0.94	1.00	0.65	0.077	59.25	13.67	0.01	0.0084	III vs. V 3.41	III vs. V 0.006
**II. Sciences**	1.04	1.00	0.62	0.078	15.13					
**III. Medicine**	1.04	1.00	0.69	0.028	09.41					
**IV. Engineering**	1.05	1.00	0.66	0.078	22.12					
**V. Social sciences**	0.88	1.00	0.64	0.037	11.43					

*only significant.

Table S62. The results of acceptance on the field of study for women.

Women										
Acceptance										
**Field of study**	**Mean**	**Median**	**SD**	**SE**	**R**	**Kruskal– Wallis H test**	**Bonferroni correction**	***p*-value**	**z-value***	**Multiple comparisons* (*p*-value)**
**I. Arts and humanities**	2.10	2.00	0.77	0.097	48.59	30.82	0.01	0.00001	II vs. III 2.92<br>III vs. V 4.84	II vs. III 0.04<br>III vs. V 0.00001
**II. Sciences**	1.97	2.00	0.84	0.096	81.31					
**III. Medicine**	2.26	2.50	0.67	0.028	32.89					
**IV. Engineering**	2.17	2.00	0.69	0.077	70.78					
**V. Social sciences**	2.03	2.00	0.75	0.046	94.02					

*only significant.

Table S63. The results of religion on the field of study for women.

Field of study	Mean	Median	SD	SE	R	Kruskal– Wallis H test	Bonferroni correction	*p*-value	z-value*	Multiple comparisons* (*p*-value)
Women										
Religion										
**I. Arts and humanities**	0.82	0.50	1.01	0.11	785.66	22.49	0.01	0.0002	III vs. V 4.00	III vs. V 0.0006
**II. Sciences**	0.65	0.00	0.91	0.10	702.99					
**III. Medicine**	0.92	0.50	1.02	0.03	824.87					
**IV. Engineering**	0.75	0.00	0.97	0.10	747.03					
**V. Social sciences**	0.67	0.00	0.91	0.05	710.18					

*only significant.

Table S64. The results of self-blame on the field of study for women.

Field of study	Mean	Median	SD	SE	R	Kruskal– Wallis H test	Bonferroni correction	*p*-value	z-value*	Multiple comparisons* (*p*-value)
Women										
Self-blame										
**I. Arts and humanities**	0.75	0.50	0.72	0.08	774.27	12.95	0.01	0.01	III vs. V 2.90	III vs. V 0.04
**II. Sciences**	0.89	0.50	0.71	0.08	872.71					
**III. Medicine**	0.78	0.50	0.69	0.02	806.75					
**IV. Engineering**	0.70	0.50	0.70	0.07	746.67					
**V. Social sciences**	0.67	0.50	0.68	0.04	723.57					

*only significant.

Table S65. The results of active coping on the field of study for men.

Field of study	Mean	Median	SD	SE	R	Kruskal– Wallis H test	Bonferroni correction	*p*-value	z-value*	Multiple comparisons* (*p*-value)
Men										
Active coping										
**I. Arts and humanities**	0.92	0.50	0.86	0.15	278.10	9.60	0.01	0.0477	IV vs. V 2.84	IV vs. V 0.04
**II. Sciences**	0.81	0.50	0.90	0.25	257.46					
**III. Medicine**	0.99	1.00	0.83	0.04	296.27					
**IV. Engineering**	0.80	1.00	0.73	0.07	260.18					
**V. Social sciences**	1.13	1.00	0.77	0.09	329.47					

*only significant.
